# The polypeptide biophysics of proline/alanine‐rich sequences (PAS): Recombinant biopolymers with PEG‐like properties

**DOI:** 10.1002/bip.23069

**Published:** 2017-10-27

**Authors:** Joscha Breibeck, Arne Skerra

**Affiliations:** ^1^ Lehrstuhl für Biologische Chemie, Technische Universität München 85354 Freising (Weihenstephan) Germany; ^2^ XL‐protein GmbH, Lise‐Meitner‐Str. 30 85354 Freising Germany; ^3^ Present address: Institut für Biophysikalische Chemie, Universität Wien 1090 Wien Austria

**Keywords:** biomimetics, hydrodynamic volume, PASylation, recombinant polypeptide, viscosity

## Abstract

PAS polypeptides comprise long repetitive sequences of the small *L*‐amino acids proline, alanine and/or serine that were developed to expand the hydrodynamic volume of conjugated pharmaceuticals and prolong their plasma half‐life by retarding kidney filtration. Here, we have characterized the polymer properties both of the free polypeptides and in fusion with the biopharmaceutical IL‐1Ra. Data from size exclusion chromatography, dynamic light scattering, circular dichroism spectroscopy and quantification of hydrodynamic and polar properties demonstrate that the biosynthetic PAS polypeptides exhibit random coil behavior in aqueous solution astonishingly similar to the chemical polymer poly‐ethylene glycol (PEG). The solvent‐exposed PAS peptide groups, in the absence of secondary structure, account for strong hydrophilicity, with negligible contribution by the Ser side chains. Notably, PAS polypeptides exceed PEG of comparable molecular mass in hydrophilicity and hydrodynamic volume while exhibiting lower viscosity. Their uniform monodisperse composition as genetically encoded polymers and their biological nature, offering biodegradability, render PAS polypeptides a promising PEG mimetic for biopharmaceutical applications.

## 1 INTRODUCTION

Poly‐ethylene glycol (PEG) is an uncharged chemical polymer with broad application in biotechnology, biophysics, nanotechnology and biomedical research, not only as a common precipitant for proteins^1^ and nucleic acids^2^ but also for hydrophilic coating of particles or surfaces, preparation of hydrogels^3^ and, in particular, for conjugation to biopharmaceuticals.^4^, ^5^ Prominent features of PEG are its strong hydrophilicity, its disordered conformation in a dissolved state, which causes an expanded hydrodynamic volume as well as “crowding effect,” the availability in a variety of average sizes and its largely inert chemical and biological behavior.^6^

During the past two decades, PEG has seen increasing use in the development of therapeutic proteins and peptides with the goal to extend their intrinsically short circulation in blood or to reduce immunogenicity.^4^ When covalently conjugated to a pharmaceutically active compound, long PEG chains—typically 20–40 kDa—with their expanded radius of gyration slow down renal filtration through the glomerular pores and, thus, can extend plasma half‐life by up to one order of magnitude.^7^ However, despite a successful history with more than 10 approved PEGylated protein/peptide drugs to date, there is an increasing awareness of drawbacks associated with PEGylation such as the high cost of clinical grade activated PEG derivatives, the extra effort for chemical coupling and, notably, the accumulation of PEG in organs and tissues upon prolonged administration at higher doses.^8^

As an alternative, we have developed a novel class of biodegradable polymers based on the natural *L*‐amino acids Pro, Ala, and Ser.^9^ These Pro/Ala‐rich sequences (PAS) show high solubility in aqueous buffers and exhibit natively disordered conformation, in particular absence of detectable secondary or tertiary structure, thus inherently mimicking the polymer behavior of PEG. Furthermore, such polypeptides can be genetically encoded and biosynthesized directly in conjunction with a therapeutic protein or peptide as fusion partner.

Indeed, several PASylated biopharmaceuticals have been generated up to now, for example growth hormone,^9^ leptin,^10^ antibody fragments,^9^, ^11^ interferons,^9^, ^12^, ^13^ erythropoietin,^14^ a complement inhibitor,^15^ and interleukin‐1 receptor antagonist (IL‐1Ra). All these PASylated proteins have demonstrated beneficial properties:^8^ (i) high solubility and stability, (ii) full biofunctionality both *in vitro* and in animal models, (iii) high bioavailability when injected subcutaneously, intraperitoneally or intramuscularly and (iv) drastically prolonged plasma half‐life—by one to two orders of magnitude—depending on the length of the PAS chain (typically, 600 residues). Moreover, PAS biopolymers are fully uncharged in a physiological environment, thus leaving the pI of an associated therapeutic component unaffected. This makes PASylation compatible with transmembrane transport processes and also ensures high productivity in widely applied expression systems such as *E. coli*, yeast and mammalian cell culture, including efficient secretion into the culture medium.^16^

Having previously demonstrated applicability of PASylation technology for biopharmaceutical drug development, we were interested in studying the inherent properties of these novel biopolymers and their relation to polymer length as well as amino acid sequence and composition. Here, we report on the extensive characterization of the hydrodynamic and biophysical properties of various PAS polymers and their comparison to PEG of different lengths. To this end, we have prepared the PAS polypeptides both as isolated biopolymer substances and as fusion proteins with IL‐1Ra, an approved biological drug (anakinra) that antagonizes interleukin‐1β.^17^ While showing prospects for the treatment of several autoinflammatory syndromes, e.g. rheumatoid arthritis and diabetes, the potential of IL‐1Ra has not been fully exploited owing to its very short plasma half‐life.

## 2 EXPERIMENTAL

### 2.1 Preparation of fluorescein‐PEGs, PAS biopolymers and PAS‐IL‐1Ra fusion proteins

PEGs with average molecular masses of 10550, 20732, 30032, and 42244 Da were purchased as α‐amino ethyl‐ω‐methoxy‐polyoxyethylenes (NOF Corporation, Tokyo, Japan) and labeled with 5(6)‐carboxyfluorescein *N*‐hydroxysuccinimide ester (Pierce Biotechnology, Rockford, IL) to enable spectrophotometric detection.

The synthetic genes for most PAS sequences used in this study were either obtained by ligation of short hybridized oligodeoxynucleotide pairs encoding repetitive amino acid sequence units according to a previously described strategy^18^ or as assembled longer synthetic DNA fragments with minimized repeat on the nucleotide sequence level.^8^ The PAS polypeptides were produced in the cytoplasm of *E. coli* KS272 as C‐terminal fusion proteins with *E. coli* thioredoxin (TrxA; UniProt ID: P0AA25). An Arg residue was inserted directly preceding the PAS#1 sequence, thus providing a trypsin proteolysis site, whereas in case of the Ser‐free P/A polypeptides a Met residue at the equivalent position permitted liberation by cyanogen bromide cleavage.^19^ Fusion proteins of human IL‐1Ra (UniProt ID: P18510) with different PAS sequences were produced in a similar way in the cytoplasm of *E. coli* Origami B using a cloned cDNA; this strain was chosen to ensure formation of the single disulfide bond in the biologically active protein.

After mechanical cell disruption and centrifugation of the extract the supernatants containing the TrxA‐PAS fusion proteins were heated to 70°C to precipitate host proteins and subsequently isolated by fractionated (NH_4_)_2_SO_4_ precipitation. Purification to homogeneity was accomplished using anion exchange chromatography (AEX). Following site‐specific cleavage according to one of the techniques mentioned above, a final subtractive AEX chromatography step, thus capturing the cleaved TrxA protein as well as traces of residual uncleaved fusion protein, yielded the pure free PAS polypeptide. UV absorption spectra were measured at 205 nm, hence probing the backbone peptide groups.^20^ The PAS‐IL‐1Ra fusion proteins were purified as soluble proteins from the raw cell extract by immobilized metal ion affinity chromatography (IMAC) on Ni Sepharose High Performance (GE Healthcare, Freiburg, Germany) and cation exchange chromatography (CEX).

### 2.2 Biochemical and biophysical characterization of PAS polypeptides and PAS‐IL‐1Ra fusion proteins

PAS polypeptides and their fusion proteins were initially characterized by ESI‐MS in the positive ion mode as well as SDS‐PAGE using either Coomassie Brilliant Blue staining or, for the free PAS polymers, a modified BaI_2_ staining procedure as originally described for PEG.^21^ For quantification of hydrophobicity, a 1 mL Resource RPC column (GE Healthcare) was used and an acetonitrile concentration gradient from 2 to 80% (v/v) in water containing 0.1% (v/v) formic acid was applied. CD spectra were measured in 50 mM K_2_SO_4_, 5 mM KP_i_ pH 7.5 with a J‐810 spectropolarimeter (Jasco, Groß‐Umstadt, Germany). Size exclusion chromatography (SEC) was performed on a Superdex S200 10/300 GL column (GE Healthcare) in 50 mM NH_4_HCO_3_, 500 mM NaCl. Viscosity was measured at 25°C with an *m*‐VROC microviscometer (Rheosense, San Ramon, CA) using a dilution series ranging from 1 to 100 mg/mL for each of the PAS and PEG samples. The concentration‐dependent viscosity data were evaluated according to Huggins^22^ and to Kraemer.^23^ After that, the Mark‐Houwink‐Sakurada parameters were extracted from both viscometry^24^ and SEC data.^25^, ^26^ DLS measurements were performed with a Zetasizer Nano‐S instrument (Malvern Instruments, Herrenberg, Germany) and the resulting parameters were evaluated according to Einstein and Simha,^27^ Stokes‐Einstein^28^ and Perrin,^29^ thus allowing shape estimation of the PAS polypeptide ensembles as prolate ellipsoids. By combining Einstein‐Simha and Mark‐Houwink‐Sakurada relationships, a direct correlation between viscosity and hydrodynamic radius was derived, validating the comparison of viscosity properties for the investigated polymers.

Detailed description of the cloning, bacterial expression and purification procedures for both TrxA and IL‐1Ra fusion proteins as well as characterization and biophysical data evaluation is given in the online Supporting Information.

## 3 RESULTS AND DISCUSSION

### 3.1 Preparation of PAS polymers and analysis via SDS‐PAGE

**Figure 1:**
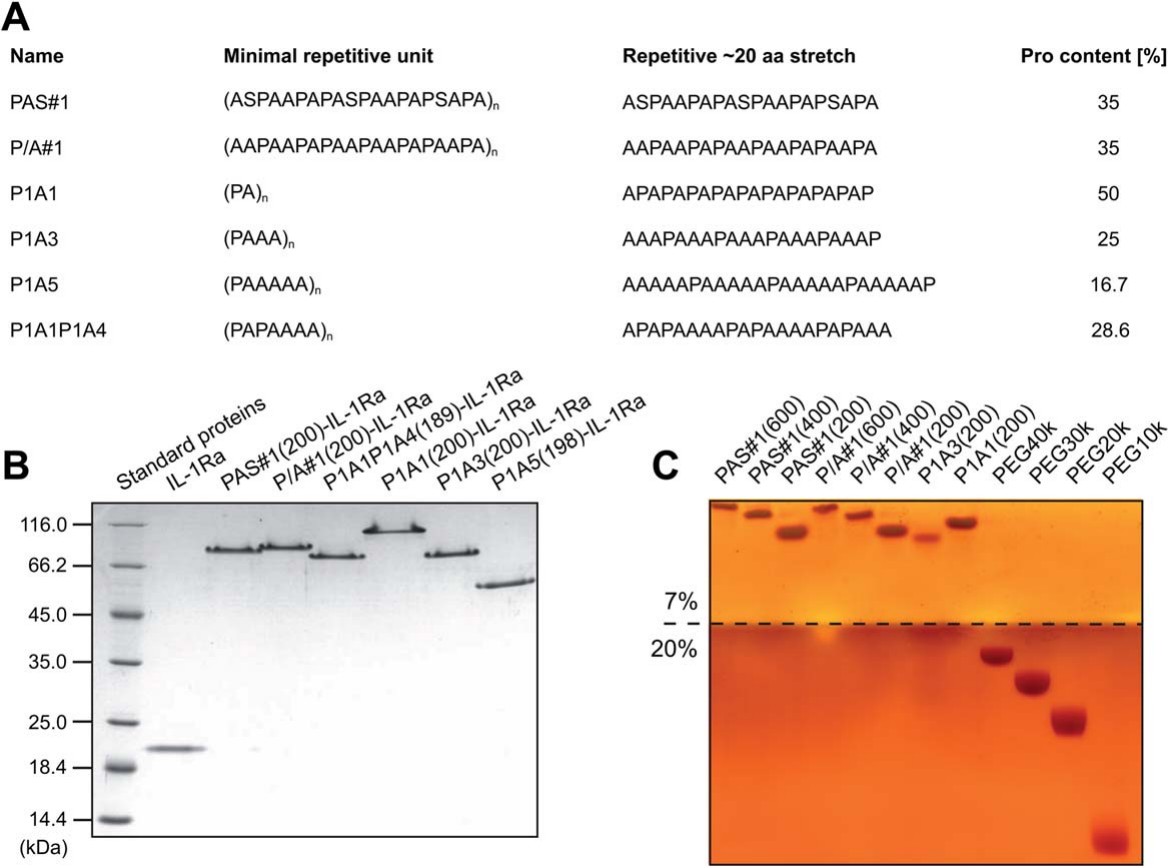
PAS polypeptides and their analysis by SDS‐PAGE. **(A)** Amino acid repeat sequences of PAS polypeptides investigated in this study and their percentages of Pro content. **(B)** SDS‐PAGE (12% (w/v) polyacrylamide gel^60^ stained with Coomassie Brilliant Blue) of various PAS‐IL‐1Ra fusion proteins. **(C)** Isolated PAS polypeptides, obtained after cleavage of TrxA‐PAS fusion proteins, in comparison with PEG polymers, both stained with BaI_2_ (7% (w/v) polyacrylamide stacking gel and 20% (w/v) running gel, borderline highlighted)

Originally, PASylation technology was devised on the basis of a repetitive sequence comprising Pro, Ala and Ser residues.^9^ Motivated by the pronounced solubility of these natively disordered polypeptides in water—despite complete lack of charged side chains—we became interested in the role of the only polar side chain present, the Ser hydroxyl group. Therefore, we derived Pro/Ala‐only (P/A) sequences from the previously described PAS#1 pattern by substituting each Ser residue by Ala, resulting in a second‐generation biopolymer dubbed P/A#1 (Figure 1). In the present study the PAS#1 and P/A#1 polypeptides were investigated and compared in three different lengths of 200, 400 and 600 residues each. Furthermore, the Pro:Ala ratio and sequence was varied in order to evaluate effects on the polymer properties in aqueous solution while maintaining a constant length (here, approximately 200 residues): P1A1, P1A3, P1A5, and P1A1P1A4 (cf. Figure 1A).

All these PAS polypeptides were produced in the cytoplasm of *E. coli* either fused to the C‐terminus of *E. coli* thioredoxin or to the N‐terminus of mature human IL‐1Ra and purified from the total cell extract by AEX. The TrxA fusion proteins were designed with a specific processing site, a Met or an Arg residue, respectively, between both moieties, thus yielding free PAS polypeptide upon chemical (cyanogen bromide) or proteolytic (trypsin) cleavage from the protein carrier followed by AEX purification. The predicted molecular masses of all polypeptide species prepared and investigated here were confirmed by ESI‐MS (Supporting Information Table S1).

First, the purified recombinant PAS polypeptides (with or without Ser) were investigated by discontinuous polyacrylamide gel electrophoresis in the presence of the anionic detergent Na‐dodecylsulfate (SDS‐PAGE), a routine method for protein/peptide analysis in biochemistry. Interestingly, both the isolated PAS polypeptides and their fusion proteins with IL‐1Ra and TrxA migrated at positions corresponding to much higher molecular weight than normally expected. While such a retarded electrophoretic mobility in comparison with conventional marker proteins was seen for other PASylated proteins before,^9^ it was most pronounced for the isolated polymers, which did not even leave the stacking gel (Figure 1B,C). This behavior is most likely explained by poor binding of SDS, whose negatively charged head groups provide the driving force in the electric field, probably due to the lack of any hydrophobic amino acid side chains.

Nevertheless, comparison of the different IL‐1Ra fusion proteins revealed progressively slower migration and, thus, apparently enlarged size with increasing PAS length. Furthermore, when comparing different PAS sequences comprising similar lengths of approximately 200 residues (Figure 1B), the electrophoretic migration behavior through the mesh‐like polyacrylamide matrix—which is determined both by SDS binding and by the length and stiffness of the polypeptide chain—turned out to depend mainly on the Pro content: P1A1 (50% Pro) showed the largest apparent size whereas for P1A5 (16.7% Pro) the fastest migration was detected. Notably, the proportion of Ser or the amino acid sequence pattern were less relevant.

These findings were in accordance with SDS‐PAGE analyses of the free PAS polypeptides side by side with PEG polymers of varied lengths on a BaI_2_‐stained gel (Figure 1C). Interestingly, the observation that PAS polypeptides, which do not bind the common protein dye Coomassie Brilliant Blue,^9^ can be stained using such a method typical for PEG^21^ illustrates certain chemical similarity of both polymers with regard to the complexation of Ba^2+^ ions. However, uncharged PEG polymers of comparable molecular size moved much faster through the gel than the PAS polypeptides. The most probable explanation for this is stronger binding of SDS by PEG, which indicates more pronounced hydrophobicity compared to the PAS biopolymers, a finding corroborated in the experiments described below.

### 3.2 Polymer solubility and hydrophilicity

Lower and upper critical solution temperatures above 100°C (0°C < 100°C < LCST < UCST) were reported for PEG polymers in the molecular weight range investigated here.^30^ Likewise, no solubility changes in the temperature range from 10 to 90°C were observed in turbidity measurements of the PAS polypeptides using typical aqueous buffers (data not shown). Moreover, similar to the chemical polymer PEG, the PAS polypeptides demonstrated high solubility not only in water but also in polar aprotic organic solvents; in fact, the isolated PAS polypeptides P/A#1 and PAS#1 were well soluble in dimethyl formamide (DMF), dimethyl sulfoxide (DMSO) and methanol. Conversely, no solubility was seen in less polar solvents such as ethanol, diethyl ether and acetonitrile, again reflecting the solubility behavior of PEG.

As a quantitative measure for the hydrophilicity of the individual PAS sequences, reversed phase chromatography (RPC) on a poly‐styrene matrix was performed with a concentration gradient of acetonitrile in water/formic acid (Supporting Information Table S2). Interestingly, the hydrophilicity of IL‐1Ra used as fusion partner was unaffected by the presence of any PAS sequence investigated, as judged from its essentially unchanged elution volume; only the P1A5 polypeptide led to a slight increase in hydrophobicity as indicated by the elevated acetonitrile concentration needed for elution. Notably, these results indicate that there are no relevant differences in hydrophobicity between the original PAS#1 polypeptides^9^ and their Ser‐free P/A#1 derivatives described here. Thus, the strong hydrophilicity of the PAS polymers must be a direct consequence of the solvent‐exposed peptide backbone in the absence of secondary structure, while any contribution by the hydroxyl groups of Ser side chains seems marginal.

To allow direct comparison of the purified free PAS polypeptides with conventional PEG, both polymers were labeled, either via the single N‐terminal amino group of the polypeptide or via a similar group provided by an amino‐PEG derivative, with fluorescein as a spectroscopic probe. In a direct comparison of the labeled PAS polypeptides with their underivatized counterparts it was seen that fluorescein, which constitutes a hydrophobic group, slightly retarded elution in the acetonitrile concentration gradient, thus illustrating the sensitivity of the RP‐HPLC analysis even for a minute increase in hydrophobicity of the polymer conjugate as a whole. However, from the side by side comparison of the derivatized PAS polypeptides with the fluorescein‐labeled PEG polymers it became evident that PEG is engaged in much stronger hydrophobic interactions with the polystyrene matrix. For example, the PEG(30 kDa) fluorescein conjugate showed a longer retention time by 34% than the P/A#1(400) conjugate, which has similar molecular mass. Longer PEG chains showed further increasing hydrophobicity as indicated by the gradually rising retention time (cf. Supporting Information Table S2).

### 3.3 Hydrodynamic volume effects quantified by SEC and DLS measurements

To compare the hydrodynamic volumes of isolated PAS polypeptides, also including the corresponding PAS‐IL‐1Ra fusion proteins, analytical size exclusion chromatography (SEC) and dynamic light scattering (DLS) measurements were performed. Calibration of the Superdex SEC column with a series of globular standard proteins (see Supporting Information Methods) was used to deduce apparent molecular sizes from elution volumes, as well as so‐called “mass enhancement factors” describing the ratio of apparent to calculated molecular mass.

**Figure 2:**
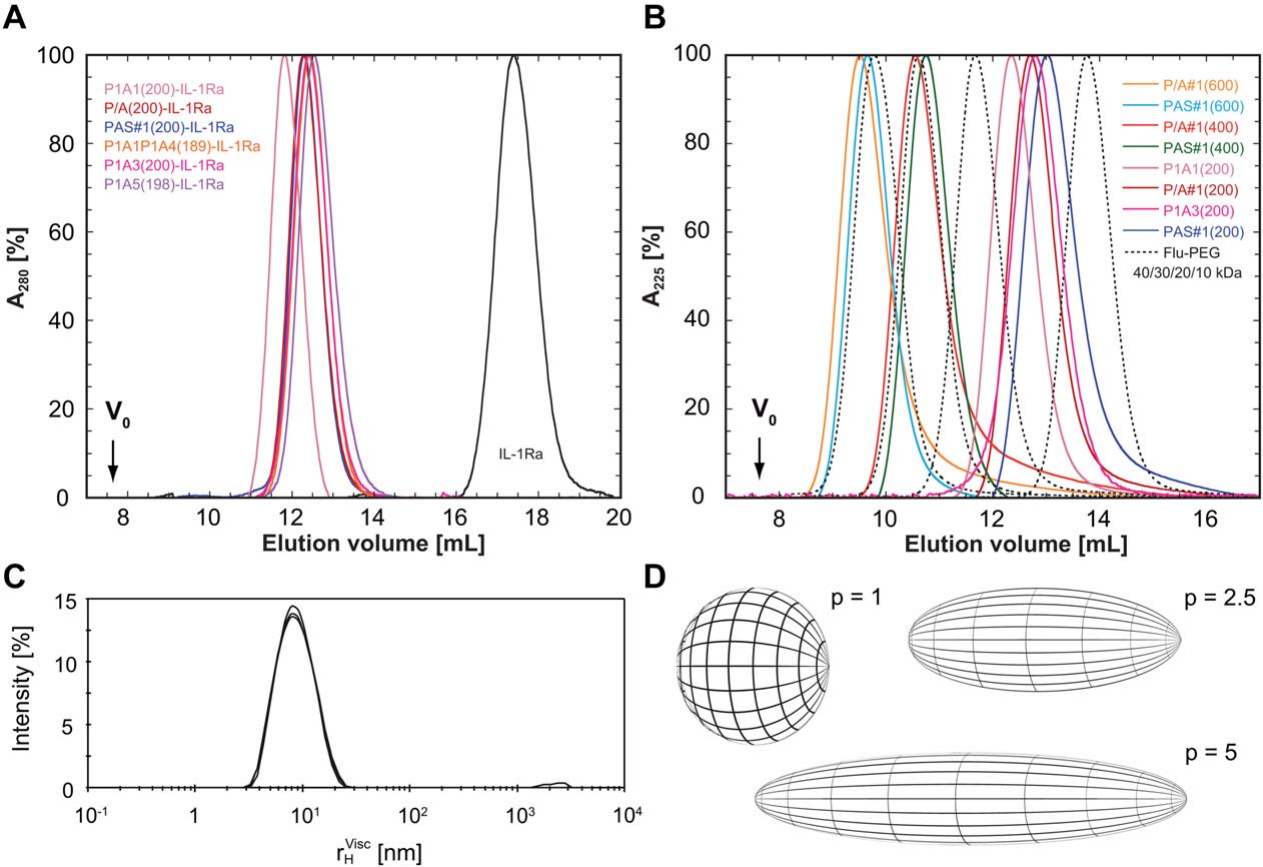
Hydrodynamic and shape properties of PAS‐IL‐1Ra fusion proteins as well as isolated PAS polypeptides investigated by SEC and DLS. **(A)** Chromatogram of IL‐1Ra and corresponding PAS fusion proteins with ∼200 residues comprising different PAS sequences. **(B)** Chromatogram of isolated (unmodified) PAS polypeptides in comparison with fluorescein‐labeled PEG polymers with varying lengths. **(C)** Exemplary *r_H_*‐correlogram (output of the DLS instrument software) for P/A#1(600) measured in triplicate by DLS, revealing monodispersity with narrow *r_H_*‐distribution. (**D)** Shape estimation of PAS polypeptides based on parameters from viscometry and DLS (see text and Supporting Information Methods). The P1A3 polypeptide and also the PEG polymers most closely match the shape of a sphere with an ideal axis ratio *p* = 1. With rising Pro content, the PAS polypeptides adopt more elongated structures corresponding to increased axis ratios. The PAS#1 and P/A#1 polypeptides with *p* = 5 are surpassed by the P1A1 polypeptide (*p* = 10, not shown), which exhibits PPII‐like structure

The SEC chromatograms of the IL‐1Ra fusion proteins with different PAS polypeptides, all comprising approximately 200 residues, revealed roughly the same elution volumes (around 12 mL; Figure 2). This indicates a massive increase in hydrodynamic molecular volume compared to plain IL‐1Ra (17.4 mL) upon PAS conjugation, in line with previous observations for other PASylated proteins and, interestingly, with only minor influence of the individual PAS sequence.^9^ The additive effect on the apparent molecular size of IL‐1Ra as a consequence of fusion with different PAS polypeptides was quantified as the ratio between measured molecular size difference and calculated mass increase due to PASylation (Δ*M*_app_/Δ*M*_calc_). In this analysis the largest ratio was found for the P1A1 sequence (11.3) and the lowest value for P1A5 (8.7). Interestingly, there was a trend toward increasing hydrodynamic volume with rising Pro content of the P/A polypeptide (Table 1).

**Table 1:** SEC elution volumes and molecular size estimation

Macromolecule	Elution volume [mL]	Calculated M^a^ [kDa]	Apparent M^b^ [kDa]	Enhancement factor for M^c^	ΔM_app_/ΔM_calc_ d	Pro content of PAS sequence [%]
IL‐1Ra	17.41	18.5	13.8	0.7	‐	‐
PAS#1(200)‐IL‐1Ra	12.29	35.1	162.1	4.6	8.9	35
P/A#1(200)‐IL‐1Ra	12.29	34.6	162.1	4.7	9.2	35
P1A1P1A4(189)‐IL‐1Ra	12.44	33.4	150.8	4.5	9.2	28.6
P1A1(200)‐IL‐1Ra	11.81	35.3	204.3	5.8	11.3	50
P1A3(200)‐IL‐1Ra	12.40	34.1	153.8	4.5	9.0	25
P1A5(198)‐IL‐1Ra	12.54	33.5	143.7	4.3	8.7	16.7
PAS#1(200)	13.02	16.6	114.1	6.9	‐	35
PAS#1(400)	10.75	33.1	340.4	10.3	‐	35
PAS#1(600)	9.65	49.6	578.3	11.7	‐	35
P/A#1(200)	12.69	16.1	133.7	8.3	‐	35
P/A#1(400)	10.55	32.2	374.9	11.6	‐	35
P/A#1(600)	9.51	48.2	618.6	12.8	‐	35
P1A3(200)	12.86	15.6	123.2	7.9	‐	25
P1A1(200)	12.34	16.9	158.3	9.4	‐	50
Fluorescein‐PEG(10k)	13.77	10.9	79.5	7.3	‐	‐
Fluorescein‐PEG(20k)	11.67	21.1	218.6	10.4	‐	‐
Fluorescein‐PEG(30k)	10.61	30.4	364.2	12.0	‐	‐
Fluorescein‐PEG(40k)	9.78	42.6	543.2	12.8	‐	‐

a Calculated with the ProtParam tool (www.expasy.org), also considering the additional mass of fluorescein where applicable.

b Experimentally determined by SEC (Superdex S200 10/300 GL, 24 mL bed volume) using globular protein size standards.

c Ratio of apparent to calculated molecular mass.

d Ratio of the apparent *versus* calculated mass increase upon PASylation of IL‐1Ra.

A similar expanded size, with elution volumes ranging from 9.5 to 13 mL, was observed in the chromatograms of the isolated PAS polypeptides, as well as for PEG polymers of different sizes (which were investigated after amino‐terminal labeling with a single fluorescein group to allow spectrophotometric detection). The free PAS#1 and P/A#1 polypeptides comprising 200 residues revealed only minor mutual differences in hydrodynamic volume, whereas the sequence P1A1 appeared larger and P1A3 showed a slightly smaller size than P/A#1, again reflecting an effect of changing Pro content.

In a complementary experimental setup, hydrodynamic radii were determined by DLS, again allowing both the estimation of apparent molecular weights and calculation of mass enhancement factors but in an independent manner from SEC (Table 2 and Figure 2C,D). Generally, the apparent molecular masses correlated well with those measured by analytical SEC. However, one should keep in mind that for the interpretation and comparison of elution volumes (or partition coefficients) from SEC, macromolecules are usually treated as spherical particles such that elongated molecular shapes lead to over‐estimation of the (apparent) molecular size.^31^, ^32^ DLS measurements, on the other hand, are even more sensitive to elongated molecular conformation.^33^ This allows determination of the ratio of the DLS‐derived apparent molecular mass to the respective SEC‐derived mass, here termed “hydrodynamic quotient” (see Supporting Information Methods).

**Table 2:** Hydrodynamic radii, molecular size and shape estimation from DLS measurements

Macromolecule	Calculated M^a^ [kDa]	Apparent M^b^ [kDa]	Enhancement factor^c^ for M	Hydrodynamic quotient^d^	$r_{H}^{\text{DLS}}$ e [nm]	[η]^f^ [ml/g]	$r_{H}^{\text{Visc}}$ g [nm]	f_P_ h (Perrin)	Prolate axial ratio p^i^	Pro content of PAS sequence [%]
IL‐1Ra	18.5	25.6	1.4	1.9	2.4	‐	‐	‐	‐	‐
PAS#1(200)‐IL‐1Ra	35.1	368.5	10.5	2.3	7.4	‐	‐	‐	‐	35
P/A#1(200)‐IL‐1Ra	34.6	303.8	8.8	1.9	6.9	‐	‐	‐	‐	35
P1A1P1A4(189)‐IL‐1Ra	33.4	224.5	6.7	1.5	6.0	‐	‐	‐	‐	28.6
P1A1(200)‐IL‐1Ra	35.3	746.5	21.1	3.7	10.1	‐	‐	‐	‐	50
P1A3(200)‐IL‐1Ra	34.1	228.0	6.7	1.5	6.1	‐	‐	‐	‐	25
P1A5(198)‐IL‐1Ra	33.5	122.7	3.7	0.9	4.7	‐	‐	‐	‐	16.7
PAS#1(200)	16.6	138.7	8.4	1.2	4.9	25.0	4.0	1.2	4.3	35
PAS#1(400)	33.1	341.3	10.3	1.0	7.2	36.6	5.8	1.2	4.8	35
PAS#1(600)	49.6	605.7	12.2	1.0	9.2	50.4	7.3	1.3	4.9	35
P/A#1(200)	16.1	138.7	8.6	1.0	4.9	24.3	4.0	1.2	4.7	35
P/A#1(400)	32.2	375.5	11.7	1.0	7.5	40.8	5.9	1.3	5.1	35
P/A#1(600)	48.2	605.7	12.6	1.0	9.2	46.2	7.1	1.3	5.7	35
P1A3(200)	15.6	86.3	5.5	0.7	4.0	33.4	4.3	0.9	‐	25
P1A1(200)	16.9	341.3	20.2	2.1	7.2	35.4	4.6	1.6	10.8	50
PEG(10k)	10.6	91.4	8.7	1.2	4.1	26.8	3.5	1.2	3.4	‐
PEG(20k)	20.7	138.7	6.7	0.6	4.9	41.8	5.1	1.0	‐	‐
PEG(30k)	30.0	278.5	9.3	0.8	6.6	51.2	6.2	1.1	2.0	‐
PEG(40k)	42.2	489.6	11.6	0.9	8.4	61.0	7.4	1.1	3.0	‐

a Calculated with the ProtParam tool (www.expasy.org), also taking into account the mass of fluorescein.

b Determined from $r_{H}^{DLS}$ data by DLS software.

c Ratio of apparent to calculated molecular weight.

d Ratio of apparent molecular masses from SEC (Table 1) and DLS analysis (this Table).

e Experimentally determined by DLS.

f Intrinsic viscosity, extrapolated from concentration‐dependent viscosity values (Supporting Information Figure S3).

g Calculated from intrinsic viscosity assuming a spherical molecular shape using the Einstein‐Simha relationship (Supporting Information Methods, equation 9).

h Perrin friction factor, calculated from $r_{H}^{DLS}$/ $r_{H}^{Visc}$ (Supporting Information Methods, equation 24).

i Calculated from *f_P_* (with DLS instrument software; cf. Supporting Information Methods, equation 25).

The hydrodynamic quotient obtained for the PAS‐IL‐1Ra fusion proteins was interpreted as a measure of shape and was close to 1 only for the P1A5 construct; hence, this PAS sequence appears to adopt the most spherical shape—as would be expected for a true random chain^34^—even in fusion with a folded globular domain (IL‐1Ra). Interestingly, with increasing Pro content this shape factor was found to increase, with a figure of 2 for PAS#1 and P/A#1, reaching the highest value of 2.3 for P1A1, thus indicating an increasingly elongated, nonglobular shape of the disordered, fluctuating polypeptide chain. When investigating the DLS data for the isolated PAS polypeptides, and also for the PEG polymers, a shape factor of roughly 1 was found for all polymers—with the only exception of the P1A1 polypeptide, which showed a value of 2.1 and thus seems to exhibit an elongated molecular conformation as isolated polymer.

For the longer P/A#1(600) polypeptide, which showed the equivalent biophysical behavior in SEC and DLS studies (see below), DLS measurements were also performed with variation of temperature (Supporting Information Figure S1). Interestingly, a moderate temperature‐dependent length increase of the random chain ellipsoid was detected in this experiment, indicating a thermally induced structural transition toward the elongated PPII conformation as will be discussed further below.

### 3.4 Polymer viscometry

**Figure 3:**
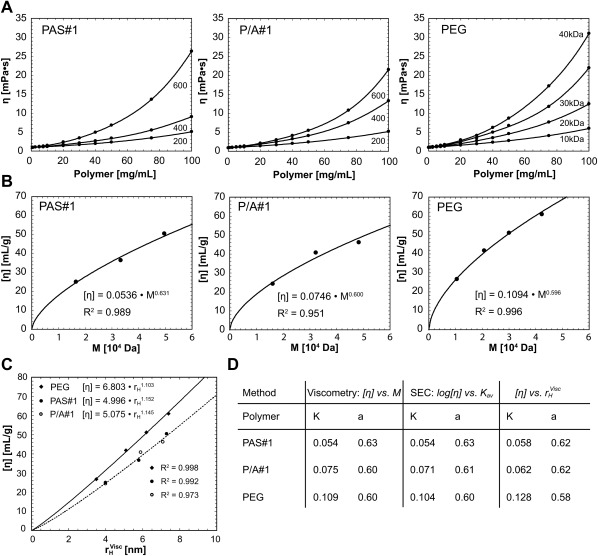
Viscosity of PAS polypeptides in comparison with PEG in aqueous solution. **(A)** Plots of viscosity against the sample concentration for PAS#1, P/A#1 and PEG with varied lengths at 25°C (fitted by a polynomial function, see Supporting Information Methods). **(B)** Evaluation of the Mark‐Houwink parameters from *[η] = K · M^a^* for the three different polymers shown in **(A)**. **(C)** Plot of *[η]* against the calculated hydrodynamic radius $r_{H}^{\mathrm{Visc}}$ for the three polymers. The intrinsic viscosity of both PAS polypeptides *versus* hydrodynamic volume shows almost the same slope, whereas PEG exhibits higher viscosity for similar $r_{H}^{Visc}$. **(D)** Mark‐Houwink‐Sakurada parameters evaluated from viscosity and SEC data using 3 different mathematical approaches (see text and Supporting Information Methods)

Microviscosity measurements were performed in aqueous solution at varying dilution for the free PAS polypeptides with different sequences and lengths and, for comparison, with PEG polymers covering the same size range. The resulting viscosity curves show the typical concentration‐dependent shape of linear hydrophilic polymers,^35^ allowing curve fitting by higher order polynomial functions. As expected, viscosities were seen to rise with increasing length of each investigated PAS sequence. Among the PAS polypeptides with ∼200 residues length, both PAS#1 and P/A#1 exhibited the lowest viscosities whereas P1A1 and P1A3 revealed higher values (Supporting Information Figure S2). Interestingly, when these data were compared to the concentration‐dependent viscosities measured for PEG with varied molecular weight, PAS polypeptides of similar size—e.g., PAS#1(600) *versus* PEG40k—always showed lower values, even at high concentrations (Figure 3).

The intrinsic viscosities *[η]* were evaluated using both Huggins^22^ and Kraemer^23^ plots (Supporting Information Figure S3), and from plots of *[η]* against the molecular mass the Mark‐Houwink^24^ parameters were extracted (Supporting Information Methods and Figure 3B). The resulting *K* and *a* parameters for the PEG samples were in the expected range.^36^, ^37^ Interestingly, for the PAS#1 and P/A#1 polypeptides the *a*‐values of 0.63 and 0.60, respectively, were slightly higher than for PEG but close to each other. This indicates random‐coil conformation in a so‐called “good” solvent,^38^ with 0.5 < *a* < 0.8. The inverse trend was seen for the *K* values, revealing a faster gain in viscosity with increasing PEG chain length than for the PAS polypeptides (Supporting Information Methods and Figure 3C,D). This finding makes PAS polypeptides a favorable alternative to the chemical PEG polymer in applications where low viscosities are desirable, for example for subcutaneous injection through thin needles.^39^

If assuming spherically shaped molecular dimensions for the conformationally disordered polymers, the hydrodynamic radii can be calculated from the intrinsic viscosity *[η]* by employing the Einstein‐Simha relationship.^28^ The ratio between the hydrodynamic radius determined by DLS (cf. above) and the resulting viscometric radius, also referred to as “Perrin friction factor,” is a measure for the deviation from the characteristic spherical shape of an ideal random chain.^29^ If deviating from 1 (i.e. the value for spherical shape), this friction factor can be physically interpreted as a prolate ellipsoid—instead of an oblate ellipsoid as alternative mathematical solution—whose elongated shape and axial proportions are in accordance with the preferred spatial extension of a linear (one‐dimensional) polypeptide chain. Interestingly, when comparing the different PAS sequences, increasing prolate axial ratios were observed with rising Pro content (Table 2). P1A3, and also the PEG polymers, most closely approximated spherical shape, whereas the PAS#1 and P/A#1 polypeptides appeared to adopt a slightly elongated average conformation while still resembling a random chain. In contrast, the P1A1 polypeptide obviously showed by far the most pronounced elongation, in line with its stiff conformational properties as will be discussed below.

A correlation of the viscometric data with the SEC elution volumes (see Supporting Information Methods) was derived by introducing the Mark‐Houwink parameters from the corresponding exponential relationship^24^ into the SEC calibration equation.^26^ The resulting linear plots of both *logM* and *log[η] versus* the chromatographic partitioning coefficient, *K_av_*, for the PAS#1 and P/A#1 polypeptides as well as PEG polymers of different lengths (Supporting Information Figure S4) offered a second and independent means to extract the parameters *K* and *a* compared with the purely viscometric analysis described above. Furthermore, by applying the Einstein‐Simha equation in combination with the Mark‐Houwink‐Sakurada equation, a direct relationship between *[η]* and the hydrodynamic radius determined by viscometry was derived (see Supporting Information Methods), thus eliminating the dependence of *[η]* on the molecular mass for each polymer, PAS#1, P/A#1 and PEG. In this third approach the *K* and *a* parameters were deduced from the plots of *[η]* against $r_{H}^{Visc}$ (Figure 3C), yielding values close to the numbers determined before from combination of the viscosity and the SEC data (Figure 3D).

### 3.5 Conformational properties deduced from CD spectroscopy

**Figure 4:**
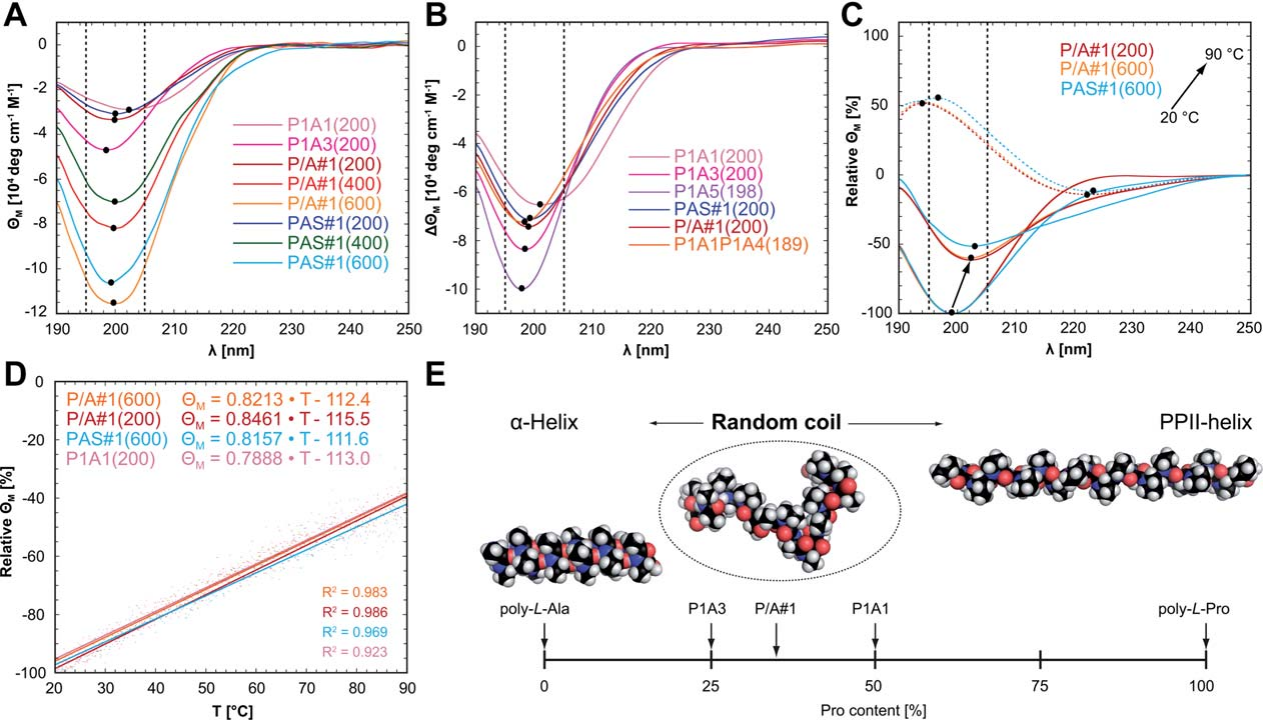
CD spectroscopy of PAS polypeptides. **(A)** CD spectra of purified PAS polypeptides. The characteristic wavelength window between 195 and 205 nm is indicated by dashed black lines, band minima (or maxima) are marked by black dots. **(B)** Difference CD spectra obtained for the IL‐1Ra fusion proteins after subtraction of the Il‐1Ra spectrum. **(C)** CD spectra (solid lines) of P/A#1(200), P/A#1(600) and PAS#1(600) at 20°C (set to ‐100%) and 90°C, together with the difference spectra between both temperatures (dashed lines). Independent of the length, all PAS polypeptides lose contributions from random‐coil structure and gain PPII structure at elevated temperatures (cf. Supporting Information Table S4). **(D)** Relative change in molar ellipticity for P/A#1(200), P/A#1(600), PAS#1(600), and P1A1(200) upon heating from 20 to 90°C with a linear temperature gradient of 1 K/min monitored at 195 nm (fitted by a straight line). **(E)** Illustration of the impact of Pro proportion on the ensemble conformation of P/A polypeptides (for explanation, see text). Zero Pro content corresponds to pure poly‐*L*‐alanine, which is known to adopt α‐helical conformation. Beyond a critical proportion of Pro residues P/A polypeptides adopt a random coil conformation with a three‐dimensionally expanded, almost spherical average appearance. However, with rising Pro content the molecular shape becomes increasingly elongated and, finally, the pure poly‐*L*‐proline chain exhibits full PPII conformation in aqueous solution

Far UV circular dichroism (CD) spectra of the isolated PAS polypeptides and their IL‐1Ra fusion proteins were recorded in the wavelength range from 190 to 250 nm at 20°C. The measured CD spectra of the pure PAS polypeptides (Figure 4) each revealed a single strong negative band around 200 nm without any positive signal, which is indicative of random coil conformation.^40^ When correcting the spectra of the PAS‐IL‐1Ra fusion proteins by subtracting the CD spectrum of the isolated IL‐1Ra protein, all recorded under the same conditions, difference spectra with the same characteristics as for the respective free PAS polypeptides were obtained (Figure 4B), similarly as described for other PASylated proteins before.^9^ Thus, the solution structure of PAS polypeptides is essentially unaffected by conjugation to another macromolecule. Also, within each series of PAS polypeptides with the same repetitive sequence (PAS#1 or P/A#1) the CD signal intensity was linearly dependent on the polypeptide length (200, 400 or 600 residues).

When comparing the different PAS polypeptides with ∼200 residues, signal intensity of the negative band tended to slightly decrease with rising Pro content (Supporting Information Figure S5a), starting with P1A5. At the same time the minimum shifted to higher wavelengths, both pointing to the Pro content as the main determinant of band shape and position, as particularly evident for P1A1. Notably, going from P1A1 over P/A#1 and PAS#1 as well as P1A3 to P1A5, the contribution of random coil structure increased as judged according to the precise wavelength of the minimum approaching the ideal value at 195 nm.^41^ On the other hand, no indication of α‐helical conformation was found with increasing Ala proportion in spite of the well documented preference of poly(*L*‐Ala) for this secondary structure.^42^ Notably, the broadened minimum seen for P1A1, shifted toward a longer wavelength of 205 nm, hinted at a detectable component from polyproline(II) (PPII) structure.^43^

It is well known that poly(*L*‐Pro) can adopt two characteristic conformations depending on solvent polarity.^43^ The right‐handed PPI helix involves all‐*cis*‐configuration of the backbone peptide bonds and is favored in a nonpolar environment such as methanol. In this contracted structure the polar polypeptide backbone is shielded by the exposed side chain rings. Conversely, the PPII structure, which prevails in aqueous buffers, is more elongated, leading to a solvent‐exposed polypeptide backbone with all‐*trans*‐configuration. Importantly, the φ‐ and ψ‐angles are almost equal for both Pro helix types, which reflects the conformational constraints conveyed on the backbone by the pyrrolidine ring of this imino acid. The known CD spectrum of poly(*L*‐Pro) in aqueous solution at pH 7.0 resembles the typical random‐coil spectrum of denatured polypeptides with a prominent negative band, yet at a longer wavelength of 205 nm.^43^

Interestingly, in the CD spectra of the PAS sequences the absence of a weak positive maximum around 225 nm, which was often seen in other studies for polypeptides in random‐coil conformation having more average amino acid composition,^44^ is reminiscent of the “unordered poly‐proline structure” described by Tiffany and Krimm^40^ that is characterized by a strong minimum around 200 nm only (Supporting Information Table S3). Taking into consideration that repetitive amino acid sequences are unlikely to adopt an ideal random conformation without any (at least locally) regular structure,^45^ it would be plausible that PAS polypeptides show a kind of random‐coil structure influenced by the PPII conformation.^46^ Of note, it has been proposed that the PPII conformation represents the energetically most favored conformation of the peptide bond,^47^ since solvation of the hydrophilic polypeptide backbone is maximized while putative entropy loss due to formation of secondary structure with more pronounced order (such as α‐helix) is kept minimal. Indeed, a considerable contribution by PPII conformation has been demonstrated for short unstructured peptides composed of Ala^48^ and Lys^49^ and was also proposed in a theoretical study.^50^

Pro, with its fixed φ angle, is also known to impose considerable conformational constraint upon the preceding amino acid within the peptide chain. Schimmel and Flory^51^ postulated that an individual *trans*‐prolyl‐residue is equally compatible—in aqueous solution—with two secondary structures: PPII and α‐helix. These considerations are in line with the observation that in folded globular proteins Pro only occurs at the beginning of an α‐helix but otherwise destabilizes this secondary structure.^52^ Accordingly, the P1A1 sequence should exhibit a stronger PPII character since each Ala residue tends to assume the PPII conformation from the following Pro. Indeed, such a tendency was detected in NMR studies of the Pro‐rich N‐terminus of the light chain of skeletal myosin,^53^ which contains a (PA)_7_ motif that exhibits an elongated conformation with the diffusion behavior of a rigid cylinder.

The possible switch of an individual *trans*‐prolyl residue within a (poly)peptide chain from PPII to the more compact α‐helical conformation can lead to a sharp turn, as found e.g. in myoglobin.^54^ Consequently, even polypeptides dominated by PPII structure are prone to disorder, which can favor random‐coil conformation to some extent,^51^ whereas *cis*/*trans*‐isomerization of peptide bonds is not involved in this mechanism. Nevertheless, the latter type of configurational isomerization, which accounts for on average 20% fluctuating *cis*‐prolyl peptide bonds,^34^ constitutes an additional source of structural disorder most likely relevant for PAS polypeptides. In fact, with increasing chain length—and number of Pro residues—this fundamental phenomenon is expected to gain significance. Unfortunately, most previous structural investigations in this context were limited to short peptides accessible by chemical synthesis^55^ and the only study devoted to higher molecular weight Pro/Ala‐rich polypeptides was based on synthetic amino acid copolymers comprising a diverse mixture of sequences and lengths.^56^ Employing genetically defined PAS biopolymers as a viable model for long polypeptide chains with regularly spaced Pro residues, the conformational effects described here provide a more comprehensive picture of disordered polypeptides in the absence of interacting side chains.

Finally, when monitoring the CD signal of different PAS sequences at 195 nm while heating the sample between 20 and 90°C (at 1 K/min), no cooperative transition was observed, thus confirming the absence of any secondary or tertiary structure. However, there was a gradual shift of the negative random coil band from 199 to 202 nm for PAS#1 and P/A#1 and from 202 to 206 nm for P1A1, corresponding to a subtle change from distorted random coil to a more PPII‐like state (as also evidenced by the 90°C *versus* 20°C difference spectra), independent of the chain length (Figure 4C,D, Supporting Information Figure S5b, Table S4). Taken together, the CD data support the assumption that the PPII helical conformation constitutes an inherent structural component of Pro‐rich polypeptides^51^ but requires sufficiently high Pro content to be observable in aqueous solution. Nevertheless, in accordance with textbook knowledge, Pro generally acts as a secondary structure breaker both by the conformational constraints imposed by its cyclic side chain and by the lack of an amide proton as hydrogen bond donor.

## 4 CONCLUSIONS

We have investigated the biophysical properties of PAS polypeptides, which constitute a novel class of artificial biopolymers with defined lengths and sequences composed of the small *L*‐amino acids Pro, Ala, and/or Ser. The hydrodynamic properties of the PAS#1 polypeptide with a 20mer repeat sequence, which represents the most widely applied PAS polymer up to now and was studied here both as isolated substance and in fusion with the exemplary biopharmaceutical IL‐1Ra, are in agreement with previous findings:^9^ PAS#1 represents a strongly hydrophilic and structurally disordered polypeptide with expanded hydrodynamic volume that closely resembles PEG and offers attractive features for the pharmacokinetic modulation of therapeutic proteins and peptides.

Interestingly, when all three Ser residues in the 20mer repeat were replaced by Ala, which led to the P/A#1 sequence, the aforementioned properties of the polypeptide were fully retained. At first glance, this result was unexpected and almost contradictory to textbook knowledge, since the removal of the hydrophilic hydroxyl side chains should lead to an overall more hydrophobic polymer. However, when taking into account the complete exposure of all backbone peptide groups (with their carbonyl H‐bond acceptors and amide H‐bond donors), it appears plausible that the contribution of the few Ser side chains to the overall hydrophilicity of the polypeptide is marginal. We have pointed out before^9^ that the PAS#1 sequence exhibits a low molar ratio between apolar hydrocarbon and polar groups, with CH_n_:(NH/CO/OH) = 1.25; in fact, this ratio is just slightly elevated for the P/A#1 sequence with 1.35. On the other hand, this figure is significantly higher for PEG, with a CH_2_:O ratio of 2. Indeed, our comparative RPC measurements provide experimental proof that PAS polypeptides in general are more hydrophilic than this well known and widely applied chemical polymer.

Evidently, the PAS polypeptides owe their pronounced hydration to the effective exposure of their backbone peptide groups to the aqueous solvent. This is only possible in the absence of regular secondary structure, which involves the extensive intramolecular pairing of H‐bond donors and acceptors in folded proteins. Indeed, the α‐helical poly‐Ala peptides, already starting from about 10 residues, are known to be insoluble in water due to their lack of available H‐bonding groups, which necessitates the incorporation of highly polar side chains to enable solution studies.^57^ Consequently, the question arises which ratio of Pro to Ala is necessary to favor a natively disordered polypeptide structure in the absence of chaotropic reagents based on the notion of Pro playing a role as “helix‐breaker.” As mentioned above, pure poly‐Ala forms a stable α‐helix in aqueous solution whereas pure poly‐Pro adopts its peculiar PPII conformation, with high solubility at all lengths,^58^ while both exhibiting a one‐dimensionally more or less elongated structure (Figure 4E). In contrast, our findings suggest that an appropriate mixture of these two amino acids leads to annihilation of their distinct inherent secondary structure propensities, thus yielding random coil conformation with an expanded hydrodynamic volume in accordance with the fundamentals of polymer biophysics.^59^

How the Pro content mechanistically affects the conformation and solubility of PAS polypeptides, can be understood as follows (Figure 4E). For long poly(*L*‐Ala) chains, which show elongated α‐helical conformation in aqueous buffers, the engagement of all hydrogen donor and acceptor groups in an intramolecular H‐bonding network hampers hydration and solubility. Once a critical proportion of Pro residues is incorporated into the sequence, thus interrupting the regular H‐bond pattern and introducing local structural perturbations as explained above, the consecutive Ala stretches become too short to stabilize α‐helical conformation. This leads to a random‐coil polypeptide conformation with a three‐dimensionally expanded, almost spherical average shape. However, with rising content of Pro residues, more stretches can adopt local PPII conformation, again resulting in an increasingly elongated shape. Finally, the pure poly(*L*‐Pro) chain exhibits full PPII conformation in aqueous solution, which lacks any intramolecular hydrogen bonds and shows strong solvation as well as excellent solubility, yet with an extended one‐dimensional shape.^43^

Indeed, the combined findings from SEC, DLS, CD and viscosity measurements indicate that the P/A#1 sequence (representing a 20mer repeat) as well as the more regular P1A3 polypeptide (having a shorter 4mer repeat sequence), with Pro content of 35 and 25%, respectively, most closely resemble an ideal random chain, and the polymer ensemble on average adopts almost spherical shape. If the Pro content gets higher (P1A1: 50% Pro), the polymer conformation tends to resemble the stiff PPII secondary structure, whereas with higher proportion of Ala (P1A5: 16% Pro) it approaches an elongated α‐helix. More detailed interpretation of the data suggests that randomly distributed PPII structural patterns introduced by the *trans*‐prolyl residues contribute to the random coil character of the PAS polypeptides, which can be described as “unordered poly‐proline structure.” On top of that, the energetically favored *cis*/*trans*‐isomerization of the prolyl‐peptide bond also has to be taken into account, which enhances the overall structural disorder of long PAS polypeptides even further.

The biochemical and biophysical characterization of PAS polypeptides has revealed astonishing similarity to the chemical PEG polymer in several aspects, in particular the high solubility in water as well as polar organic solvents and the disordered, expanded random chain behavior. However, PAS biopolymers show larger hydrodynamic volume than PEG polymers of the same molecular mass but lower concentration‐dependent viscosity. The latter constitutes a benefit for therapeutic administration of corresponding drug conjugates via injection. In this context, the more pronounced hydrophilicity of PAS polypeptides used as alternative to PEG also explains the high bioavailability that has been seen in a series of animal experiments upon subcutaneous or intramuscular injection of PASylated proteins.^9^, ^10^, ^15^ Finally, due to their genetically encoded nature PAS polypeptides offer the features of biodegradability (especially via lysosomal proteases/peptidases), site‐specific chemical modification (through the amino‐terminus, the carboxy‐terminus or via purposely introduced side chains, e.g. Cys) and of strict monodispersity (as demonstrated by the extremely simple mass spectra). With regard to chemical conjugation, the P/A sequences, which lack any side chain reactivities and exhibit just a single nucleophilic N‐terminal amino group, appear particularly useful. Hence, these novel biopolymers offer numerous applications in medicine and biotechnology.

## CONFLICT OF INTEREST

A.S. is cofounder and shareholder of XL‐protein GmbH, the company that commercializes PASylation technology.

## Supporting information

Additional Supporting Information may be found online in the supporting information tab for this article.

Supporting Information

## References

[1] K. C. Ingham , Methods Enzymol. 1990, 182, 301. 2314243 10.1016/0076-6879(90)82025-w

[2] K. R. Paithankar , K. S. Prasad , Nucleic Acids Res. 1991, 19, 1346. 2030954 10.1093/nar/19.6.1346 PMC333871

[3] D. Hutanu , M. D. Frishberg , L. Guo , D. C. C , Mod. Chem. Appl. 2014, 2, 132.

[4] P. L. Turecek , M. J. Bossard , F. Schoetens , I. A. Ivens , J. Pharm. Sci. 2016, 105, 460. 26869412 10.1016/j.xphs.2015.11.015

[5] A. Kolate , D. Baradia , S. Patil , I. Vhora , G. Kore , A. Misra , J. Control. Release 2014, 192, 67. 24997275 10.1016/j.jconrel.2014.06.046

[6] E. Markovsky , H. Baabur‐Cohen , A. Eldar‐Boock , L. Omer , G. Tiram , S. Ferber , P. Ofek , D. Polyak , A. Scomparin , R. Satchi‐Fainaro , J. Control. Release 2012, 161, 446. 22286005 10.1016/j.jconrel.2011.12.021

[7] G. Pasut , F. M. Veronese , Adv. Drug Deliv. Rev. 2009, 61, 1177. 19671438 10.1016/j.addr.2009.02.010

[8] U. Binder , A. Skerra , Curr. Opin. Colloid Interface Sci. 2017, 31, 10.

[9] M. Schlapschy , U. Binder , C. Börger , I. Theobald , K. Wachinger , S. Kisling , D. Haller , A. Skerra , Protein Eng. Des. Sel. 2013, 26, 489. 23754528 10.1093/protein/gzt023 PMC3715784

[10] V. Morath , F. Bolze , M. Schlapschy , S. Schneider , F. Sedlmayer , K. Seyfarth , M. Klingenspor , A. Skerra , Mol. Pharm. 2015, 12, 1431. 25811325 10.1021/mp5007147

[11] C. T. Mendler , L. Friedrich , I. Laitinen , M. Schlapschy , M. Schwaiger , H. J. Wester , A. Skerra , mAbs 2015, 7, 96. 25484039 10.4161/19420862.2014.985522 PMC4622060

[12] D. Harari , N. Kuhn , R. Abramovich , K. Sasson , A. L. Zozulya , P. Smith , M. Schlapschy , R. Aharoni , M. Koster , R. Eilam , A. Skerra , G. Schreiber , J. Biol. Chem. 2014, 289, 29014. 25193661 10.1074/jbc.M114.602474 PMC4200257

[13] E. A. Zvonova , A. V. Ershov , O. A. Ershova , M. A. Sudomoina , M. B. Degterev , G. N. Poroshin , A. V. Eremeev , A. P. Karpov , A. Y. Vishnevsky , I. V. Goldenkova‐Pavlova , A. V. Petrov , S. V. Ruchko , A. M. Shuster , Appl. Microbiol. Biotechnol. 2017, 101, 1975. 27833991 10.1007/s00253-016-7944-3

[14] M. H. Hedayati , D. Norouzian , M. Aminian , S. Teimourian , R. Ahangari Cohan , S. Sardari , M. R. Khorramizadeh , Protein J. 2017, 36, 36. 28168382 10.1007/s10930-017-9699-9

[15] N. Kuhn , C. Q. Schmidt , M. Schlapschy , A. Skerra , Bioconjugate Chem. 2016, 27, 2359. 10.1021/acs.bioconjchem.6b00369 27598771

[16] S. D. Cesare , U. Binder , T. Maier , A. Skerra , Bioprocess Int. 2013, 11, 30.

[17] C. A. Dinarello , Annu. Rev. Immunol. 2009, 27, 519. 19302047 10.1146/annurev.immunol.021908.132612

[18] M. Schlapschy , I. Theobald , H. Mack , M. Schottelius , H. J. Wester , A. Skerra , Protein Eng. Des. Sel. 2007, 20, 273. 17595342 10.1093/protein/gzm020

[19] E. Gross , Methods Enzymol. 1967, 11, 238.

[20] R. K. Scopes , Protein Purification: Principles and Practice, 3rd ed, Springer, New York 1994.

[21] B. Skoog , Vox Sang. 1979, 37, 345. 44395 10.1111/j.1423-0410.1979.tb02314.x

[22] M. L. Huggins , J. Am. Chem. Soc. 1942, 64, 2716.

[23] E. O. Kraemer , Ind. Eng. Chem. 1938, 30, 1200.

[24] I. Sakurada , A. Nakajima , O. Yoshizaki , K. Nakamae , Kolloid‐Z.u.Z.Polymere 1962, 186, 41.

[25] Z. Grubisic , P. Rempp , H. Benoit , J. Polym. Sci. B 1967, 5, 753.

[26] G. Callec , A. W. Anderson , G. T. Tsao , J. E. Rollings , J. Polym. Sci. A 1984, 22, 287.

[27] R. Simha , J. Phys. Chem. 1940, 44, 25.

[28] A. Einstein , Ann. Phys. (Berlin) 1905, 322, 549.

[29] F. Perrin , J. Phys. Radium 1934, 5, 497.

[30] J. Seuring , S. Agarwal , Macromolecules 2012, 45, 3910.

[31] P. L. Dubin , J. M. Principi , Macromolecules 1989, 22, 1891.

[32] E. T. McGuinness , J. Chem. Educ. 1973, 50, 826. 4759054 10.1021/ed050p826

[33] J. M. Schurr , K. S. Schmitz , Ann. Rev. Phys. Chem. 1986, 37, 271.

[34] T. E. Creighton , Proteins: structures and molecular properties, 2nd ed., W.H. Freeman, New York 1992.

[35] R. Pamies , J. Hernández Cifre , M. del Carmen López Martínez , J. García de la Torre , Colloid Polym. Sci. 2008, 286, 1223.

[36] F. E. Bailey , J. L. Kucera , L. G. Imhof , J. Polym. Sci. A 1958, 32, 517.

[37] P. Gregory , M. B. Huglin , Makromol. Chem. 1986, 187, 1745.

[38] M. A. Masuelli , J. Polym. Biopolym. Phys. Chem. 2014, 2,

[39] P. Nichols , L. Li , S. Kumar , P. M. Buck , S. K. Singh , S. Goswami , B. Balthazor , T. R. Conley , D. Sek , M. J. Allen , mAbs 2015, 7, 212. 25559441 10.4161/19420862.2014.985504 PMC4622976

[40] M. L. Tiffany , S. Krimm , Biopolymers 1968, 6, 1767. 5704346 10.1002/bip.1968.360061212

[41] M. L. Tiffany , S. Krimm , Biopolymers 1969, 8, 347.

[42] D. Shental‐Bechor , S. Kirca , N. Ben‐Tal , T. Haliloglu , Biophys. J. 2005, 88, 2391. 15653741 10.1529/biophysj.104.050708 PMC1305339

[43] S. Kakinoki , Y. Hirano , M. Oka , Polym. Bull. 2005, 53, 109.

[44] S. Y. Venyaminov , I. A. Baikalov , Z. M. Shen , C. S. C. Wu , J. T. Yang , Anal. Biochem. 1993, 214, 17. 8250221 10.1006/abio.1993.1450

[45] C. Y. Hayashi , N. H. Shipley , R. V. Lewis , Int. J. Biol. Macromol. 1999, 24, 271. 10342774 10.1016/s0141-8130(98)00089-0

[46] T. Lefèvre , J. Leclerc , J. F. Rioux‐Dubé , T. Buffeteau , M. C. Paquin , M. E. Rousseau , I. Cloutier , M. Auger , S. M. Gagné , S. Boudreault , C. Cloutier , M. Pézolet , Biomacromolecules 2007, 8, 2342. 17658884 10.1021/bm7005517

[47] I. Gokce , R. W. Woody , G. Anderluh , J. H. Lakey , J. Am. Chem. Soc. 2005, 127, 9700. 15998070 10.1021/ja052632x

[48] R. V. Pappu , G. D. Rose , Protein Sci. 2002, 11, 2437. 12237465 10.1110/ps.0217402 PMC2373714

[49] A. L. Rucker , T. P. Creamer , Protein Sci. 2002, 11, 980. 11910041 10.1110/ps.4550102 PMC2373527

[50] J. A. Vila , H. A. Baldoni , D. R. Ripoll , A. Ghosh , H. A. Scheraga , Biophys. J. 2004, 86, 731. 14747311 10.1016/S0006-3495(04)74151-X PMC1303923

[51] P. R. Schimmel , P. J. Flory , J. Mol. Biol. 1968, 34, 105. 5760450 10.1016/0022-2836(68)90237-4

[52] R. Aurora , G. D. Rose , Protein Sci. 1998, 7, 21. 9514257 10.1002/pro.5560070103 PMC2143812

[53] D. G. Bhandari , B. A. Levine , I. P. Trayer , M. E. Yeadon , Eur. J. Biochem. 1986, 160, 349. 3769935 10.1111/j.1432-1033.1986.tb09978.x

[54] J. C. Kendrew , H. C. Watson , B. E. Strandberg , R. E. Dickerson , D. C. Phillips , V. C. Shore , Nature 1961, 190, 666. 13752474 10.1038/190666a0

[55] H. J. Dyson , P. E. Wright , Annu. Rev. Biophys. Biophys. Chem. 1991, 20, 519. 1867725 10.1146/annurev.bb.20.060191.002511

[56] Y. Iizuka , C. Uchida , K. Wakamatsu , M. Oya , Bull. Chem. Soc. Jpn. 1993, 66, 1269.

[57] J. M. Scholtz , R. L. Baldwin , Annu. Rev. Biophys. Biomol. Struct. 1992, 21, 95. 1525475 10.1146/annurev.bb.21.060192.000523

[58] A. Berger , J. Kurtz , E. Katchalski , J. Am. Chem. Soc. 1954, 76, 5552.

[59] C. R. Cantor , P. R. Schimmel , Biophysical Chemistry, 2nd ed, W.H. Freeman, New York 1980.

[60] S. P. Fling , D. S. Gregerson , Anal. Biochem. 1986, 155, 83. 3454661 10.1016/0003-2697(86)90228-9

