# Supplementary material for: The polypeptide biophysics of proline/alanine‐rich sequences (PAS): Recombinant biopolymers with PEG‐like properties
Source: Biopolymers. 2017 Oct 27;109(1):e23069. doi: 10.1002/bip.23069 (PMC5813227; doi:10.1002/bip.23069)
Supplement: Supplementary file 1 — Supporting Information [file BIP-109-na-s001.pdf]

SUPPORTING INFORMATION

The polypeptide biophysics of proline/alanine-rich sequences (PAS): recombinant biopolymers with PEG-like properties

Joscha Breibeck[1†] & Arne Skerra[1,2*]

[1] Lehrstuhl für Biologische Chemie, Technische Universität München, 85354 Freising (Weihenstephan), Germany

[2] XL-protein GmbH, Lise-Meitner-Str. 30, 85354 Freising, Germany

[†]Current position: Institut für Biophysikalische Chemie, Universität Wien, 1090 Wien, Austria

*Corresponding author:

Prof. Dr. Arne Skerra, Lehrstuhl für Biologische Chemie, Technische Universität München, 85354 Freising (Weihenstephan), Germany;
phone: +49 8161 71 4351; fax: +49 8161 71 4352; e-mail: skerra@tum.de

SUPPORTING INFORMATION METHODS

Polyethylene glycol samples and fluorescent labelling

The polyethylene glycol (PEG) samples used in this study were purchased from NOF Corporation (Tokyo, Japan) as α-amino ethyl-ω-methoxy polyoxyethylenes with average molecular weights of 10550, 20732, 30032 and 42244 Da, respectively. Solutions of 150 mg/mL were prepared in 50 mM $NaHCO_3$ (Roth, Karlsruhe, Germany) pH 8.0 for subsequent experiments.

For fluorescent labelling, PEG samples at concentrations of 1 mM in the buffer mentioned above were incubated with a 2fold molar amount of 5-(and 6-)carboxy fluorescein *N*-hydroxy succinimidyl (NHS) ester (Pierce Biotechnology, Rockford, IL), dissolved in dry dimethylformamide (DMF; Roth, Karlsruhe, Germany), for 1 h at room temperature. The reaction mixture was applied to a PD-10 column (GE Healthcare, Freiburg, Germany) equilibrated with 50 mM $NaHCO_3$ pH 8.0 in order to separate the labelled product from reagents prior to further analysis.

Cloning of PAS gene cassettes and fusion proteins

The synthetic genes for the PAS polypeptides used in this study were obtained by assembly of double-stranded oligodeoxynucleotide cassettes encoding an appropriate repetitive unit of each PAS sequence according to a previously described strategy.[1] After phosphorylation using polynucleotide kinase (PNK; Fermentas, St. Leon-Rot, Germany), pairs of complementary oligodeoxynucleotides (Thermo Fisher Scientific, Ulm, Germany) were hybridized, leading to mutually complementary non-palindromic 5'-GCC/GGC-overhangs (encoding Ala) at both sides. These short gene cassettes were ligated in the presence of a limiting amount of T4 DNA ligase (Fermentas) to effect partial ligation to concatamers. The corresponding PAS gene fragment of about 600 base pairs (encoding 200 amino acids) was extracted from a preparative agarose gel and subcloned on a derivative of pUC19[2] carrying in its multiple cloning region a double (inverse repeat) recognition sequence for the type IIS

restriction enzyme *Sap*I (5'-GCTCTTCN'NNN,NGAAGAGC), thus allowing DNA sequence analysis and precise gene excision from the amplified plasmid. For some of the PAS sequences, synthetic genes with less repetitive nucleotide sequence[3] were kindly provided by XL-protein, Freising, Germany. The different PAS polypeptide sequences investigated in this study are shown in Fig. 1.

The *E. coli* thioredoxin gene (*trxA*; encoding amino acid residues 2-109, UniProt ID P0AA25) was initially amplified from genomic DNA via PCR and cloned on the vector pASK37,[4] including an N-terminal His_6-tag. To allow for in frame insertion of a PAS gene cassette and subsequent cleavage of the fusion protein by cyanogen bromide, a Met codon as well as a *Sap*I recognition site were appended to the C-terminus via PCR using the primers 5'-CCACTCCCTATCAGTGAT-3' and 5'-CTCGACGCTAACCTGGCCATGGCCTGAAGAGCAA GCTTGACCTG-3'. The PCR product was digested with *Xba*I and *Hin*dIII and ligated with the correspondingly cut plasmid pASK37. An internal Met residue (at position 45 in TrxA) was replaced by Gln via QuikChange site-directed mutagenesis (Stratagene, La Jolla, CA) using the primer pair 5'-GTGCGGTCCGTGCAAACAGATCGCCCCGATTCTG-3' and 5'-CAGAATCGGGGCGATCTGTTTGCACGGACCGCAC-3'. Following insertion of an appropriate (preferably Ser-free) PAS gene cassette at the *Sap*I cleavage site, the modified TrxA protein can be separated as one intact fragment from the encoded TrxA-PAS fusion protein via cyanogen bromide (BrCN) cleavage at the single Met residue between both moieties. In case of TrxA fusion proteins with the PAS#1 polypeptide, this Met residue was exchanged by Arg in order to introduce a tryptic cleavage site using the QuikChange primer pair 5'-GCTAACCTGGCCCGTGCCTGAAGAGCAAG-3' and 5'-CTTGCTCTTCAGGCAC GGGCCAGGTTAGC-3'. Utilizing the single *Sap*I restriction site, the 600 bp gene cassette for the PAS sequence (200 amino acid residues) was then inserted, if necessary repeatedly in a successive manner (potentially with varying nucleotide sequences), to yield longer inserts of 1200 and 1800 bp, encoding 400 and 600 amino acid residues, respectively.

The genes for the PAS-IL-1Ra fusion proteins were obtained in a similar manner. To this end, the IL-1Ra gene was amplified via PCR from a cloned cDNA (RZPD, Berlin, Germany)

with primers 5'-ACGATCGGCGCCAGCTCTTCTGCCCGACCCTCTGGGAGAAAATCC-3' and 5'-CTGGGCAAGCTTACTCGTCCTCCTGGAAGTAG-3'. After restriction enzyme digest, the PCR product was inserted into the multiple cloning region of the vector pASK-IBA4 (IBA, Göttingen, Germany) via its *Kas*I and *Hin*dIII sites. During this step, also an N-terminal *Sap*I recognition site was introduced. To obtain the final construct for cytoplasmic expression in *E. coli*, the IL-1Ra gene was subcloned via *Sap*I and *Hin*dIII onto a derivative of the vector pASK75[5] which carried a T7 ribosomal binding site but did not encode an OmpA signal sequence. The PAS gene cassette was subsequently inserted via the unique *Sap*I restriction site at the N-terminus of IL-1Ra, hence allowing direct expression of corresponding fusion proteins with an N-terminal His_6-tag.

Expression and purification of PAS fusion proteins

The PAS-IL-1Ra fusion proteins were obtained in a soluble state via cytoplasmic expression in *E. coli* Origami B.[6] This strain is known for a less reducing milieu in its cytoplasm, thus permitting formation of energetically favored disulfide bonds, here the single structural disulfide bond in IL-1Ra.[7] Bacteria were cultivated in 2 L LB medium in shake flasks at 30 °C and induced at OD ≈ 1 for 4 h with 200 µg/L anhydrotetracycline (aTc; Acros Organics, Geel, Belgium). After harvest by centrifugation, the bacterial pellet was homogenized in a French pressure cell (SLM-Aminco, Urbana, IL) and clarified by centrifugation as well as sterile filtration (Filtropur, 0.45 µm; Sarstedt, Nürnbrecht, Germany). The resulting protein solution was loaded onto a Ni/NTA resin (Ni Sepharose High Performance; GE Healthcare, Freiburg, Germany) and eluted with a concentration gradient from 0 to 150 mM imidazole/HCl in 500 mM NaCl, 40 mM NaP_i pH 7.5. After analysis by SDS-PAGE, suitable fractions were combined and dialyzed against 40 mM NH_4OAc/AcOH pH 6.5. This step effected precipitation of most of the remaining host cell proteins, which could be removed by centrifugation. After application of the supernatant to a 1 mL Resource S cation exchange column (GE Healthcare), elution in a concentration gradient from 0 to 300 mM NaCl in the same buffer served for final purification.

The TrxA-PAS fusion proteins were produced in a soluble state in the cytoplasm of *E. coli* KS272.[8] Bacteria were grown at 25 °C in an 8 L bench top fermenter in M9 glucose minimal medium using a previously published protocol[9] and induced at OD_{550} ≈ 30 for 4 h with 0.5 mM isopropyl-β-D-1-thiogalactopyranoside (IPTG; Formedium, Hanstanton, UK). After harvest by centrifugation, the bacterial pellet was resuspended in about 1 L 500 mM NaCl, 40 mM NaP_i pH 7.5, homogenized in a PANDA Plus homogenizer (GEA Niro Soavi, Biberbach, Germany) and then heated to 70 °C for 15 min in a water bath to precipitate the majority of host cell proteins. After cooling to ~20 °C in a cold-room, the supernatant was clarified by centrifugation and stored frozen at -20 °C.

From the thawed solution, aggregated protein was removed by centrifugation and the supernatant was treated with 0.18 volumes (corresponding to 15 % saturation) 4.1 M $(NH_4)_2SO_4$ (AppliChem, Darmstadt, Germany) on a magnetic stirrer at 4 °C to achieve fractionated protein precipitation. Initially precipitated host proteins were separated by centrifugation and, after adding another 0.13 volumes of the $(NH_4)_2SO_4$ solution (corresponding to 25 % saturation), the PAS fusion protein was selectively precipitated and recovered by centrifugation whereas other contaminants remained in the supernatant. This protein pellet was resolubilized in 20 mM Tris/HCl pH 9.5 and dialyzed over night against the same buffer, prior to application to a 30 mL Resource 15 Q anion exchange column (GE Healthcare). Elution of pure protein was finally performed with a NaCl concentration gradient from 0 to 200 mM in the same buffer.

Preparation of PAS polymers by proteolysis or chemical cleavage

For preparative cleavage of the TrxA-PAS#1(200), TrxA-PAS#1(400) and TrxA-PAS#1(600) constructs, about 100 mg fusion protein purified via anion exchange chromatography (AEX) was digested with ~5 mg recombinant trypsin (TrypLE Select; Life Technologies, Darmstadt, Germany) at 37 °C overnight.

Alternatively, cleavage of the corresponding Ser-free P/A-polypeptides from the TrxA fusion partner was achieved by treatment with BrCN.[10] To this end, about 100 mg of the fusion protein was dialyzed three times against 50 mM NH_4HCO_3 pH 8.5 and lyophilized in a Speed Vac centrifuge (Christ, Osterode am Harz, Germany). The dried protein was dissolved at 5 mg/mL in 70 % (v/v) formic acid (Roth, Karlsruhe, Germany). Then, a 100fold molar amount of BrCN (5 M solution in DMF; Fluka, Steinheim, Germany) was added and the cleavage reaction was allowed to proceed for 1–2 days. The solution was again lyophilized and the resulting solid was resolubilized in 20 mM Tris/HCl pH 9.5 under ultrasonication for 15 min.

The raw product of each cleavage reaction was dialyzed against 20 mM Tris/HCl pH 9.5 overnight and, after sterile filtration, applied to AEX as described above. This time the uncharged PAS biopolymer appeared in the flow through and was collected, whereas residual uncleaved fusion protein as well as the liberated TrxA moiety remained bound to the column. The resulting PAS polypeptide solution was concentrated by ultrafiltration (10 kDa cut-off for PAS(400) and PAS(600), 3.5 kDa cut-off for PAS(200)).

For concentration measurement the PAS samples were diluted 1:1000 with spectroscopy buffer (50 mM K_2SO_4, 5 mM KP_i pH 7.5). After that, a 350 µL aliquot of the diluted polypeptide solution was applied to a PD-10 column pre-equilibrated with spectroscopy buffer and eluted in 3.5 mL of the same buffer, leading to a final 1:10 000 dilution with respect to the starting concentration of the PAS polypeptide. UV absorption spectra were recorded in a UV/Vis Spectrophotometer (Ultrospec 2100 pro; GE Healthcare) and quantified at 205 nm:[11]

(1) $$\varepsilon_{205}^{mg/mL} = 27 + 120 \cdot \frac{E_{280}}{E_{205}}$$

Mass spectrometry of PAS polymers

The PAS-IL-1Ra fusion proteins and the isolated PAS polypeptides were dialyzed against 10 mM NH_4OAc at a protein concentration of about 20 µM using Slide-A-Lyzer Mini devices

(MWCO 10 kDa; Thermo Fisher Scientific). For the PAS fusion proteins, solutions were supplemented with 20 % (v/v) acetonitrile (LC-MS grade; Sigma-Aldrich, Steinheim, Germany) and 0.1 % (v/v) formic acid (LC-MS grade; Sigma-Aldrich) and then analyzed on a maXis ESI Q-TOF mass spectrometer (Bruker Daltonics, Bremen, Germany). Raw data were deconvoluted with Compass Data Analysis software (version 4.0; Bruker). For the isolated PAS polypeptides, samples were supplemented with 50 % (v/v) methanol (LC-MS grade; Sigma-Aldrich) and 1 % (v/v) acetic acid (LC-MS grade; Sigma-Aldrich). In this case analysis was performed on a 6210 Time-of-Flight LC/MS instrument (Agilent Technologies, Waldbronn, Germany) and deconvoluted with MassHunter BioConfirm software (version B.03.01; Agilent).

SDS-PAGE

Fusion proteins, PAS polypeptides and PEG polymers were analyzed by SDS-PAGE using the buffer system of Fling & Gregerson.[12] PEG can be conventionally stained in SDS polyacrylamide gels using a barium iodide/iodine solution.[13-14] We found that essentially the same procedure can be applied to detect PAS polypeptides. In our modified protocol, the gel (after electrophoresis) was first rinsed with water and then incubated in 2.5 % (w/v) BaI_2 (barium iodide dihydrate; Sigma-Aldrich) for 5 min. After rinsing with water again, the gel was transferred to Lugol solution, prepared by dissolving 5 % (w/v) I_2 (Riedel de Haen, Seelze-Hannover, Germany) in 10 % (w/v) KI (AppliChem), and incubated for 5 min. Subsequent destaining in water cleared the red-brownish background to yellow and led to dark red/brown bands. Of note, the stain faded within several minutes, thus requiring immediate photographic recording.

Reverse Phase Chromatography (RPC)

Solutions of the purified PAS polypeptides and fluorescein-labelled PEG polymers with concentrations of about 1 mg/mL were first adjusted to 2 % (v/v) acetonitrile, 0.1 % (v/v)

formic acid (buffer A). Then, a 500 µl sample was applied to a 1 mL Resource RPC column (GE Healthcare) equilibrated with buffer A. Elution was subsequently achieved with a linear gradient from 100 % buffer A to 100 % buffer B (80 % (v/v) acetonitrile, 0.1 % (v/v) formic) over 20 bed volumes at a flow rate of 2 mL/min. Sample-specific UV absorption was monitored for both types of polymer at 225 nm, thus detecting either the PAS peptide bonds or the chromophore of the fluorescein-PEG conjugate. Note that the fluorescein-specific absorption around 496 nm does not prevail in the acidic milieu of the buffers applied here.

Circular dichroism spectroscopy (CD)

Secondary structure analyses were performed with a J-810 spectropolarimeter (Jasco, Groß-Umstadt, Germany) using a 0.1 mm path length quartz cuvette (106-QS; Hellma, Müllheim, Germany) and ~10 µM protein solutions in 50 mM K_2SO_4, 5 mM KP_i pH 7.5. Spectra were recorded at room temperature within a wavelength range from 190 to 250 nm by accumulating 15 runs (bandwidth 1 nm, scan speed 100 nm/min, response time 4 s). The spectra were corrected for buffer blank and smoothed using the instrument software. The molar ellipticity θ_M was calculated according to the equation

(2) $$\theta_M = \theta_{obs}/(c \bullet d)$$

where θ_{obs} is the measured ellipticity, *c* the protein concentration [mol/L] and *d* the path length of the quartz cuvette [cm]. The normalized data were plotted against the wavelength using KaleidaGraph (Synergy Software, Reading, PA). For the PAS-IL-1Ra fusion proteins, the θ_M spectrum of the unfused IL-1Ra was subtracted to obtain the difference spectrum corresponding to the PAS moiety alone. In another experiment, a linear temperature gradient from 20 to 90 °C was applied at 1 K/min and the change in ellipticity at 195 nm was monitored.

Size exclusion chromatography (SEC)

A protein sample of 300 µL was applied to a Superdex S200 10/300 GL column (GE Healthcare) equilibrated with 500 mM NaCl, 50 mM NH_4HCO_3. For IL-1Ra and its fusion proteins a sample concentration of 0.5 mg/mL was chosen and UV absorbance was recorded at 280 nm. For the isolated PAS polypeptides, sample concentrations were adjusted to 10 µg/mL and absorption of the peptide bonds was detected at 225 nm. Fluorescein-PEGs were applied at a concentration of 0.5 mg/mL and monitored both at 225 nm and at 496 nm (fluorescein-specific absorption maximum at approximately neutral pH). For estimation of apparent molecular sizes the SEC column was calibrated with a series of certified globular standard proteins (Sigma-Aldrich): cytochrome C (12.4 kDa), carbonic anhydrase (29 kDa), ovalbumin (43 kDa), bovine serum albumin (66 kDa), alcohol deydrogenase (150 kDa), β-amylase (200 kDa) and apoferritin (443 kDa).

Viscometry

Polymer viscosity was measured at 25 °C for dilution series ranging from 100 to 1 mg/mL of the PAS and PEG samples using an *m*-VROC microviscometer (Rheosense, San Ramon, CA) equipped with an mVROC2.5-GA05 flow cell. Individual viscosity data represent the average from triplicate measurements of the same sample. Generally, the dependence of viscosity on solute concentration *c* can be mathematically expressed as a Taylor series:[15]

(3) $$\eta = A_0 + A_1 c + A_2 c^2 + A_3 c^3 \ldots$$

For purposes of graphical display (see, e.g., Fig. 3a), each the lowest order (i.e., from 3 to 6) was chosen to yield an acceptable curve fit.

At reasonably low polymer concentrations, higher polynomial orders become negligible and different linearization methods with regard to *c* can be applied, e.g. as proposed by Huggins[16] (4) or, alternatively, by Kraemer[17] (5):

(4) $$\frac{\eta_{sp}}{c} = k_H [\eta]^2 \cdot c + [\eta]$$

(5) $$\frac{ln(\eta_{rel})}{c} = -k_K[\eta]^2 \cdot c + [\eta]$$

where

(6) $$\eta_{sp} = (\eta - \eta_0)/\eta_0$$

and

(7) $$\eta_{rel} = \eta/\eta_0$$

as derived from the measured sample viscosity η and the viscosity of the pure solvent η_0.

The intrinsic viscosity, [η], of the polymer solute can be obtained by extrapolation of both equations to infinitesimal concentration:

(8) $$[\eta] = lim_{c\to 0}\frac{\eta_{sp}}{c} = lim_{c\to 0}\frac{ln(\eta_{rel})}{c} = lim_{c\to 0}\frac{\eta_{rel}-1}{c}$$

Intrinsic viscosities [η] for each PAS or PEG polymer were evaluated according to both methods using concentrations of 10, 20, 30, 40, and 50 mg/mL and curve fit according to equations (4) and (5), respectively (see Fig. S3). Values for [η] were then calculated from the two linear graphs as the mean of both extrapolated axis intercepts, i.e. at zero sample concentration *c*.

To estimate the hydrodynamic radius, r_H^{Visc}, from this viscometric analysis the Einstein-Simha relation[18] can be applied to solute molecules under the assumption of a spherical shape (with mass *M* and the Avogadro number N_A):

(9) $$r_H^{Visc} = \left(\frac{3}{4\pi}\cdot\frac{[\eta]M}{2.5N_A}\right)^{\frac{1}{3}}$$

Accordingly, from the intrinsic viscosity [η] determined above (usually given in mL/g) values for r_H^{Visc} were calculated for each polymer.

Empirically, the intrinsic viscosity is known to be correlated to the average (in case of a polydisperse size mixture such as for PEG) molecular mass *M* of a polymer by the Mark-Houwink-Sakurada equation:[19]

(10) $$[\eta] = K \cdot M^a$$

with the proportionality constant K and the so-called shape factor a, which ranges from 0.5 to 1.0 for random coil polymers.

Notably, these two characteristic parameters can also be estimated independently from the SEC data for the investigated polymers (see Fig. 3d). In SEC, the elution volume V is usually considered to correlate with M as follows:[20]

(11) $$logM = -A \cdot K_{av} + B$$

A and B are constants while the K_{av} parameter relates to the measured elution volume, V, with V_0 as the void volume and V_t as the total (bed) volume of the column:

(12) $$K_{av} = \frac{V - V_0}{V_t - V_0}$$

In order to obtain a universal calibration curve valid for all kinds of polymers irrespective of their specific conformational preferences,[20-21] the intrinsic viscosity $[\eta]$ should be included as another factor in equation (11) in a more precise treatment since, in fact, it is the logarithm of the hydrodynamic volume determined by the product $[\eta]\cdot M$ (not just M) that is proportional to K_{av}:[21]

(13) $$log([\eta] \cdot M) = -A \cdot K_{av} + B$$

Usually, for molecular weight estimation of ordinary globular proteins a constant value of $[\eta]$ is assumed (typically in the range of 0.71–0.75),[22] which results in the same ordinate intercept (included in the apparent constant B) for both the protein calibrants and analytes and, therefore, its influence is neglected.

However, when investigating by SEC less compact disordered or elongated polymers, such as PEG or PAS, using a set of globular standard proteins for size calibration, there is a strong deviation in $[\eta]$ values involved (with a much higher value for $log\ ([\eta]\cdot M)$ in case of the structurally distorted polymers). This leads to an overestimation of the "apparent" molecular

weight for such polymers[23] according to the conventional equation (11), as was also applied for the ordinary SEC analysis in the present study up to this point.

If the K_{av} values for different lengths (i.e., masses *M*) of the same polymer are evaluated according to equation (11), the constants A and B – with the latter implicitly including -*log* [*η*] for this polymer according to eq. (13) – can be individually determined by linear regression for each polymer type.

On the other hand, rearrangement of equation (10) yields:

(14) $$logM = \frac{log[\eta] - logK}{a}$$

As the expressions for *log M* from equations (11) and (14) are similar, both can be combined and rearranged to a new relationship between [*η*] and the SEC elution volumes:

(15) $$\frac{log[\eta] - logK}{a} = -A \cdot K_{av} + B$$

(16) $$log[\eta] = -a \cdot A \cdot K_{av} + a \cdot B + logK$$

With the knowledge of *A* and *B* for a polymer as determined from the K_{av} measurements by SEC as explained above, a linear plot of *log* [*η*] from the corresponding viscometry data *versus* K_{av} permits independent evaluation (without explicit use of the polymer mass) of the Mark-Houwink parameters *a* (from the slope, -*a*·*A*) and *K* (from the ordinate intercept, *a*·*B* + *log K*; see Fig. 3d and Fig. S4).

Notably, the relationship between [*η*] and *M* according to equation (9), in combination with equation (10), can also be employed for alternative determination of parameters *a* and *K* using the correlation between [*η*] and the corresponding hydrodynamic radius r_H^{Visc} (with the compound constant $E = \frac{10 \cdot \pi \cdot N_A}{3}$):

(17) $$[\eta] \cdot M = E \cdot (r_H^{Visc})^3$$

However, this equation still contains the molecular mass *M*, which differs for the polymers under investigation. Applying the expression of *M* according to equation (10) from the viscometry analysis leads to:

(18) $$[\eta] \cdot \left(\frac{[\eta]}{K}\right)^{\frac{1}{a}} = E \cdot (r_H^{Visc})^3$$

Solving for [*η*] results in the final expression:

(19) $$[\eta] = F \cdot (r_H^{Visc})^G$$

with the compound parameters

(20) $$F = E^{\frac{a}{a+1}} \cdot K^{\frac{1}{a+1}}$$

and

(21) $$G = \frac{3a}{a+1}$$

which are constants for each polymer type. Thus, [*η*] solely depends on r_H^{Visc} and the Mark-Houwink shape factor *a*, beside *K*, as characteristic parameter for each polymer type independent of the molecular mass (or polymer length). This viscometric relationship can be used, as a third approach, to validate the previously found polymer parameters *a* and *K* (for summary and comparison, see Fig. 3d) that were determined, first, from viscometry by plotting [*η*] against *M* according to equation (10) and, second, from analytical SEC by plotting *log* [*η*] against K_{av} according to equation (16).

Dynamic light scattering (DLS)

IL-1Ra proteins, PAS polypeptides and unlabelled PEG samples, all at a concentration of 1 mg/mL and after microfiltration through Spin-X centrifugal units (0.45 µm; Corning Life Science, Tewksbury, MA), were applied to a 3 mm path length quartz kuvette (105.251-QS; Hellma) and the hydrodynamic radii, r_H^{DLS}, were measured at 25 °C in a Zetasizer Nano-S instrument (Malvern Instruments, Herrenberg, Germany) as average of triplicates. To

investigate the influence of temperature on the polypeptide shape, the sample P/A#1(600) was also measured after heating from 20 to 90 °C in steps of 10 K.

In this DLS technique the hydrodynamic radius r_H^{DLS} is automatically calculated by the instrument software from the translational friction factor *f* as derived from the experimentally determined diffusion coefficient *D* according to the Stokes-Einstein model:[18]

(22) $$D = \frac{k_B \cdot T}{f}$$

with

(23) $$f = 6\pi\eta r_H^{DLS}$$

Equation (22) contains the experimental r_H^{DLS} value together with the viscosity η of the dilute polymer solution, which is assumed as the one of pure water (1.00 cP at 20 °C). Notably, equation (23) is only valid for a spherical particle shape, whereas different molecular shapes lead to deviations from the linear relationship between *f* and r_H^{DLS}.

For less anisotropic, elongated molecular shapes the rotational contribution to the friction factor can no longer be neglected, which leads to enlarged experimental friction factors and, likewise, apparent hydrodynamic radii obtained by DLS.[24] For this reason, the DLS method is even more sensitive to the molecular shape than SEC (see above). Thus, a comparison of apparent molecular masses independently estimated by both techniques can be used as an empirical measure, which we have termed “hydrodynamic quotient”, for the degree of conformational elongation of a polymer chain in solution.

Perrin[25] has shown a way to estimate the shape of an ellipsoid, serving as a first approximation of an average molecular ensemble structure deviating from the ideal spherical shape in solution, by calculating the friction factor, f_P, from the ratio of the corresponding hydrodynamic radii:

(24) $$f_P = \frac{f_{Ellipsoid}}{f_{Sphere}} = \frac{f^{DLS}}{f^{Visc}} = \frac{r_H^{DLS}}{r_H^{Visc}}$$

The derivation by Perrin finally leads to two mathematical expressions for f_P that can be interpreted as prolate and oblate ellipsoids.[25-26] In the present study, only the prolate ellipsoid was considered as a reasonable shape estimation for the investigated polymers since a linear chain should exhibit a single preferred dimension of propagation:

(25) $$f_P^{prolate} = p^{\frac{2}{3}} \cdot \frac{\frac{\sqrt{p^2-1}}{p}}{atanh\frac{\sqrt{p^2-1}}{p}}$$

With an algorithm implemented in the DLS instrument software (Zetasizer Software version 6.32; Malvern Instruments) mathematical solutions for the prolate axial ratio *p*, derived from the f_P value, can be calculated from the measured r_H^{DLS} and the independently determined r_H^{Visc} parameters. Of note, instead of r_H^{Visc}, the specific volume $\bar{V}$ was the required input for the algorithm. To this end, $\bar{V}$ was calculated from [η] via the hydrodynamic volume V_H for spherical molecules (see section on viscometry) by rearrangement of equation (9) using

(26) $$V_H = \frac{4}{3}\pi(r_H^{Visc})^3$$

thus yielding:

(27) $$\bar{V} = \frac{V_H \cdot N_A}{M} = \frac{[\eta]}{2.5}$$

SUPPORTING INFORMATION REFERENCES

[1] M. Schlapschy, I. Theobald, H. Mack, M. Schottelius, H. J. Wester, A. Skerra, *Protein Eng. Des. Sel.* **2007**, *20*, 273.

[2] C. Yanisch-Perron, J. Vieira, J. Messing, *Gene* **1985**, *33*, 103.

[3] U. Binder, A. Skerra, *Curr. Opin. Colloid Interface Sci.* **2017**, *31*, 10.

[4] A. Skerra, A. Plückthun, *Protein Eng.* **1991**, *4*, 971.

[5] A. Skerra, *Gene* **1994**, *151*, 131.

[6] P. H. Bessette, F. Aslund, J. Beckwith, G. Georgiou, *Proc. Natl. Acad. Sci. U S A* **1999**, *96*, 13703.

[7] H. A. Schreuder, J. M. Rondeau, C. Tardif, A. Soffientini, E. Sarubbi, A. Akeson, T. L. Bowlin, S. Yanofsky, R. W. Barrett, *Eur. J. Biochem.* **1995**, *227*, 838.

[8] H. J. Meerman, G. Georgiou, *Biotechnology* **1994**, *12*, 1107.

[9] W. Schiweck, A. Skerra, *Proteins* **1995**, *23*, 561.

[10] E. Gross, *Methods Enzymol.* **1967**, *11*, 238.

[11] R. K. Scopes, *Protein Purification: Principles and Practice*. 3rd ed.; Springer: New York, **1994**.

[12] S. P. Fling, D. S. Gregerson, *Anal. Biochem.* **1986**, *155*, 83.

[13] M. M. Kurfurst, *Anal. Biochem.* **1992**, *200*, 244.

[14] B. Skoog, *Vox Sang.* **1979**, *37*, 345.

[15] R. Pamies, J. Hernández Cifre, M. del Carmen López Martínez, J. García de la Torre, *Colloid Polym. Sci.* **2008**, *286*, 1223.

[16] M. L. Huggins, *J. Am. Chem. Soc.* **1942**, *64*, 2716.

[17] E. O. Kraemer, *Ind. Eng. Chem.* **1938**, *30*, 1200.

[18] A. Einstein, *Ann. Phys. (Berlin)* **1905**, *322*, 549.

[19] I. Sakurada, A. Nakajima, O. Yoshizaki, K. Nakamae, *Kolloid-Z.u.Z.Polymere* **1962**, *186*, 41.

[20] G. Callec, A. W. Anderson, G. T. Tsao, J. E. Rollings, *J. Polym. Sci. A* **1984**, *22*, 287.

[21] Z. Grubisic, P. Rempp, H. Benoit, *J. Polym. Sci. B* **1967**, *5*, 753.

[22] E. T. McGuinness, *J. Chem. Educ.* **1973**, *50*, 826.

[23] P. L. Dubin, J. M. Principi, *Macromolecules* **1989**, *22*, 1891.

[24] J. M. Schurr, K. S. Schmitz, *Ann. Rev. Phys. Chem.* **1986**, *37*, 271.

[25] F. Perrin, *J. Phys. Radium* **1934**, *5*, 497.

[26] S. H. Koenig, *Biopolymers* **1975**, *14*, 2421.

SUPPORTING INFORMATION TABLES

Table S1: ESI mass-spectrometric analysis of PAS-IL-1Ra fusion proteins and isolated PAS polypeptides

	**Calc. average mass [Da]** [a]	**Observed average mass [Da]**	**Mass difference [Da]**
MKH_6-IL-1Ra	18538.9	18538.5 [b]	0.4
MKH_6-PAS#1(200)-IL-1Ra	35057.3	35056.3 [b]	1.0
MKH_6-P/A#1(200)-IL-1Ra	34577.3	34577.8 [b]	-0.5
MKH_6-P1A1P1A4(189)-IL-1Ra	33378.8	33377.8 [b]	1.0
MKH_6-P1A1(200)-IL-1Ra	35358.5	35359.2 [b]	-0.7
MKH_6-P1A3(200)-IL-1Ra	34056.6	34056.6 [b]	0
MKH_6-P1A5(198)-IL-1Ra	33471.8	33470.8 [b]	1.0
PAS#1(200)	16607.4	16607.3 [c]	0.1
PAS#1(400)	33125.8	33125.7 [c]	0.1
PAS#1(600)	49644.2	49645.1 [c]	-0.9
P/A#1(200)	16127.5	16127.5 [c]	0
P/A#1(400)	32165.9	32165.2 [c]	0.7
P/A#1(600)	48204.3	48202.3 [c]	2.0
P1A3(200)	15606.7	15606.6 [c]	0.1
P1A1(200)	16908.6	16908.5 [c]	0.1

[a] calculated with the ProtParam tool (http://www.expasy.org), also considering the loss of 2 Da in IL-1Ra due to formation of one disulfide bridge

[b] determined by ESI-MS with the maXis Q-TOF instrument

[c] determined by ESI-MS with the 6210 TOF LC/MS instrument

Table S2: Hydrophobic interactions on a polystyrene matrix determined by RPC. Chromatography of the polymers was performed on a 1 mL Resource RPC column applying an acetonitrile (ACN) gradient from 2 to 80 % (v/v) in 20 column volumes at 2 mL/min flow rate. The difference in retention time caused by the fluorescein-labelling of P/A#1 polypeptides is indicated in brackets.

	**Retention time (Δt) [min]**	**ACN for elution [%]**	**Proline content [%]**
IL-1Ra	4.46	38.04	-
PAS#1(200)-IL-1Ra	4.53	38.58	35
P/A#1(200)-IL-1Ra	4.43	37.80	35
P1A1P1A4(189)-IL-1Ra	4.45	37.96	28.6
P1A1(200)-IL-1Ra	4.56	38.74	50
P1A3(200)-IL-1Ra	4.44	37.80	25
P1A5(198)-IL-1Ra	5.22	43.81	16.7
PAS#1(200)	3.27	28.36	35
PAS#1(400)	3.37	29.14	35
PAS#1(600)	3.54	30.55	35
P/A#1(200)	3.31	28.68	35
P/A#1(400)	3.48	30.16	35
P/A#1(600)	3.55	30.56	35
P1A3(200)	3.27	28.36	25
P1A1(200)	3.37	29.14	50
Fluorescein-P/A#1(200)	3.94 (Δ = 0.63)	33.75	35
Fluorescein-P/A#1(400)	3.93 (Δ = 0.45)	33.67	35
Fluorescein-P/A#1(600)	3.91 (Δ = 0.36)	33.43	35
Fluorescein-PEG(10000)	5.49	47.32	–
Fluorescein-PEG(20000)	5.71	48.18	–
Fluorescein-PEG(30000)	5.82	48.96	–
Fluorescein-PEG(40000)	5.89	49.50	–

Table S3: Location of the characteristic random coil minimum and the corresponding molar ellipticity Θ_M in CD spectra recorded for isolated PAS polypeptides and in the difference spectra of the IL-1Ra fusion proteins ($\Delta\Theta_M$; after subtraction of the spectrum for IL-1Ra itself).

		λ_{min} [nm]	Θ_M [10^4 deg/M·cm]	Pro content [%]
PAS#1(200)	free	200.1	-3.077	35
PAS#1(400)	free	200.2	-7.018	35
PAS#1(600)	free	199.7	-11.676	35
P/A#1(200)	free	199.6	-3.336	35
P/A#1(400)	free	199.4	-8.189	35
P/A#1(600)	free	199.7	-11.567	35
P1A3(200)	free	198.7	-3.341	25
P1A1(200)	free	202.6	-2.893	50
PAS#1(200)	$\Delta\Theta_M$	199.4	-7.122	35
P/A#1(200)	$\Delta\Theta_M$	199.3	-7.431	35
P1A1P1A4(189)	$\Delta\Theta_M$	198.0	-7.239	28.6
P1A5(198)	$\Delta\Theta_M$	197.9	-10.003	16.6
P1A3(200)	$\Delta\Theta_M$	198.4	-8.376	25
P1A1(200)	$\Delta\Theta_M$	201.7	-6.515	50

Table S4: Effect of rise in temperature on the CD characteristics for PAS polypeptides of various lengths and sequences. Characteristic band positions are indicated for spectra recorded at two different temperatures and also for the corresponding difference spectra (ΔT).

	PAS#1(600)	P/A#1(600)	P/A#1(200)	P1A1(200)	P1A3(200)
**λ_{min}(20 °C) [nm]**	199.1	199.1	199.2	201.8	198.5
**λ_{min}(90 °C) [nm]**	202.7	202.0	202.2	205.9	-
**λ_{min}(ΔT) [nm]**	196.5	193.9	194.1	197.0	198.7
**λ_{max}(ΔT) [nm]**	223.4	222.4	222.3	223.7	225.7

Table S5: Dependence of observed CD signal intensities on the percentage of Pro for the different P/A polypeptide sequences under study. The molar ellipticities Θ_M from Table S3 were obtained from difference spectra between various IL-1Ra-P/A fusion proteins and the IL-1Ra protein itself. For precise comparison, the Θ_M values were normalized with respect to a reference length of exactly 200 residues. A plot of the resulting normalized intensities against the reciprocal Pro content is depicted in Fig. S5a.

	Θ_M **[10^4 deg/M•cm]**	**Pro content [%]**	Θ_M^{norm} **[10^4 deg/M•cm]** [a]	**Reciprocal Pro content [1/%]**
P1A5(198)	-10.003	16.6	-10.053	0.060
P1A3(200)	-8.376	25	-8.334	0.040
P1A1P1A4(189)	-7.239	28.6	-7.620	0.035
P/A#1(200)	-7.431	35	-7.394	0.029
P1A1(200)	-6.515	50	-6.483	0.020

[a] normalized for a polymer length of precisely 200 amino acids

SUPPORTING INFORMATION FIGURES

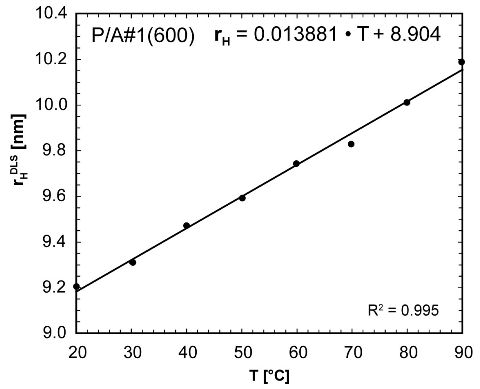

Fig. S1: Change of the hydrodynamic radius of P/A#1(600) with rising temperature measured by DLS. The data points (each obtained as average from triplicate measurements) can be fitted by a straight line and are in agreement with the linear increase in PPII structural contributions *versus* temperature as observed in the CD spectroscopy.

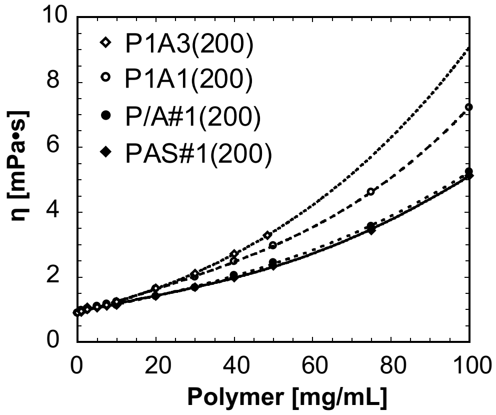

Fig. S2: Comparison of viscosities for different PAS polypeptides having the same length of 200 residues. Plots of measured solution viscosity against the sample concentration (in 20 mM Tris/HCl pH 9.5). Data were fitted by a polynomial function of higher order (here 3rd order) according to equation (3).

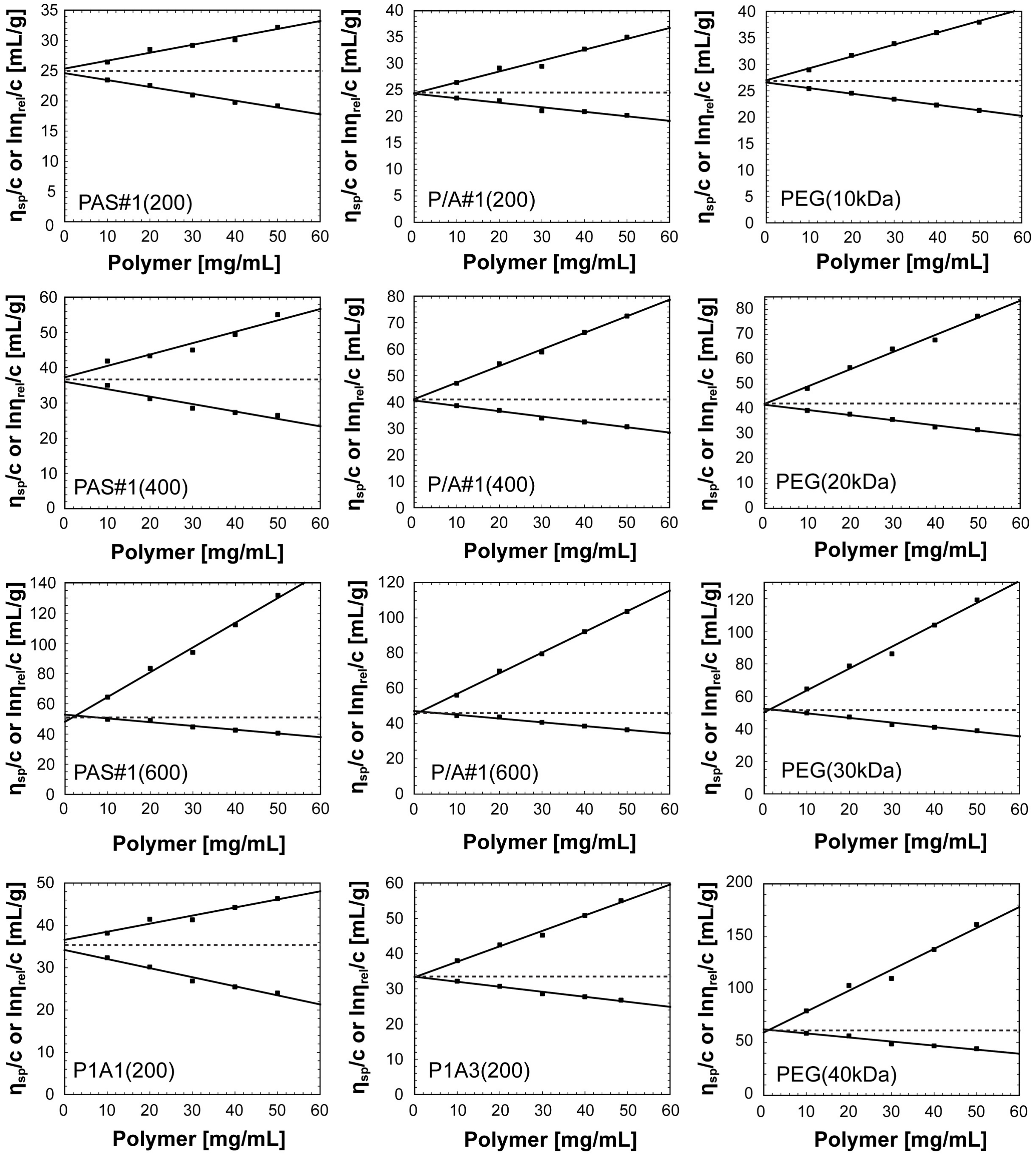

Fig. S3: Evaluation of the intrinsic viscosities [η] for the polymers investigated in this study according to Huggins and Kraemer (equations 4 and 5). The ordinate intercepts are marked by horizontal dashed lines indicating the average value from both linear extrapolations.

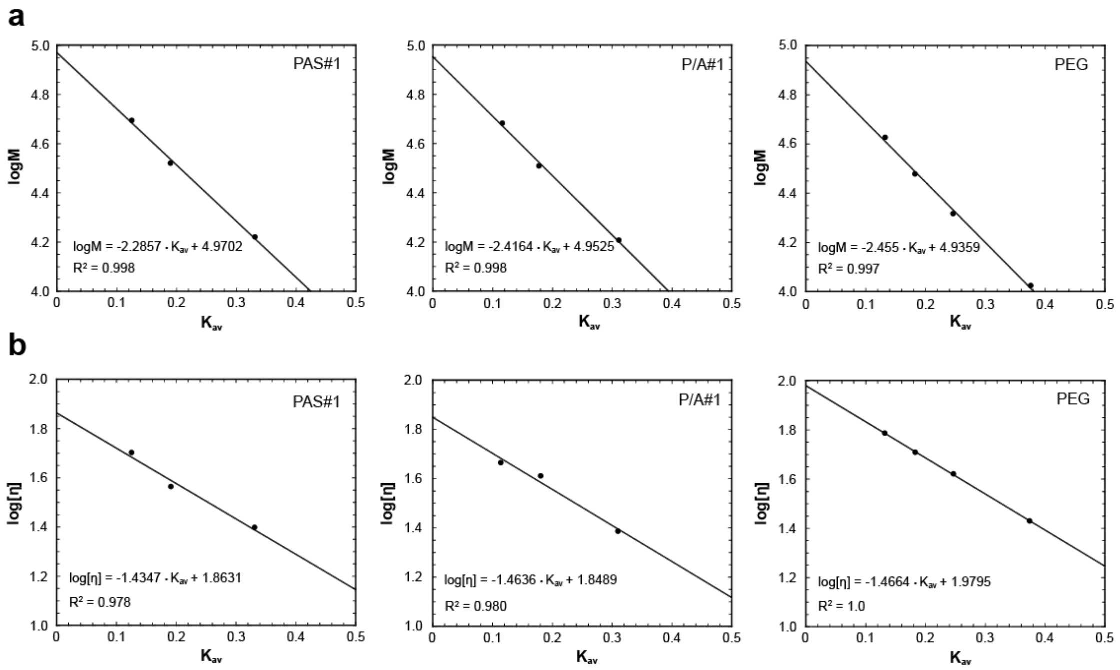

Fig. S4: Evaluation of the Mark-Houwink parameters *a* and *K* for the three polymers PAS#1, PA#1 and PEG from the SEC and viscometry data based on plots of logarithms of (**a**) the molecular weight *M* and (**b**) the intrinsic viscosity [*η*], both against K_{av} (linear regression according to equations 11 and 16, respectively). Resulting parameters are listed in Fig. 3d.

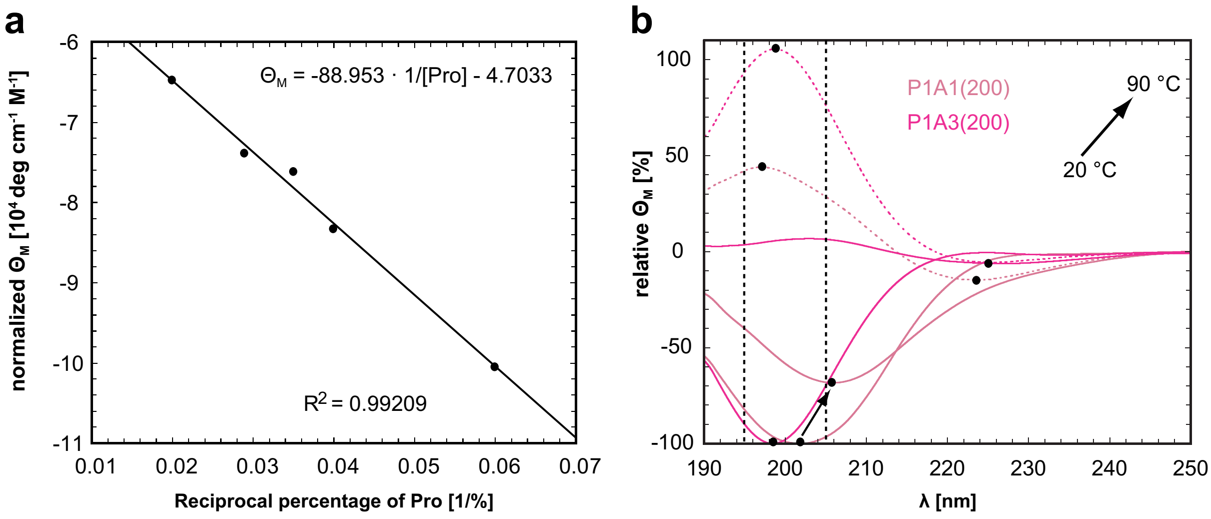

Fig. S5: CD analyses of P/A polypeptides. (**a**) Correlation between CD intensities and Pro percentage. The normalized molar ellipticities for all investigated P/A sequences with varied Pro:Ala composition reveal a linear dependence on the reciprocal Pro content, that is the negative CD band intensities around 200 nm get more pronounced with decreasing proportion of Pro (see Table S5). (**b**) CD spectra of P1A1(200) and P1A3(200) polypeptides, both at 20 and 90 °C. Spectra are shown in solid lines together with the corresponding difference spectra (dashed lines) between the two temperatures. Local intensity minima and maxima are marked with black dots in order to illustrate the spectral changes upon change in temperature. The P1A1 polypeptide loses contributions from random coil while gaining additional PPII structure at elevated temperatures (see also Table S3). In contrast, P1A3 aggregated during the heating process and yielded a CD spectrum indicating a structure dominated by β-turn.
